# PTBP1 controls intestinal epithelial regeneration through post-transcriptional regulation of gene expression

**DOI:** 10.1093/nar/gkad042

**Published:** 2023-02-06

**Authors:** Ullas Valiya Chembazhi, Wesley S Tung, Hyojeong Hwang, Yuexi Wang, Aryan Lalwani, Ka Lam Nguyen, Sushant Bangru, Danielle Yee, Kristy Chin, Jing Yang, Auinash Kalsotra, Wenyan Mei

**Affiliations:** Department of Biochemistry, University of Illinois Urbana-Champaign, Urbana, IL 61801, USA; Department of Comparative Biosciences, College of Veterinary Medicine, University of Illinois Urbana-Champaign, Urbana, IL 61802, USA; Department of Comparative Biosciences, College of Veterinary Medicine, University of Illinois Urbana-Champaign, Urbana, IL 61802, USA; Department of Comparative Biosciences, College of Veterinary Medicine, University of Illinois Urbana-Champaign, Urbana, IL 61802, USA; Department of Comparative Biosciences, College of Veterinary Medicine, University of Illinois Urbana-Champaign, Urbana, IL 61802, USA; Department of Comparative Biosciences, College of Veterinary Medicine, University of Illinois Urbana-Champaign, Urbana, IL 61802, USA; Department of Biochemistry, University of Illinois Urbana-Champaign, Urbana, IL 61801, USA; Department of Comparative Biosciences, College of Veterinary Medicine, University of Illinois Urbana-Champaign, Urbana, IL 61802, USA; Department of Comparative Biosciences, College of Veterinary Medicine, University of Illinois Urbana-Champaign, Urbana, IL 61802, USA; Department of Comparative Biosciences, College of Veterinary Medicine, University of Illinois Urbana-Champaign, Urbana, IL 61802, USA; Department of Biochemistry, University of Illinois Urbana-Champaign, Urbana, IL 61801, USA; Cancer Center at Illinois, University of Illinois Urbana-Champaign, Urbana, IL 61801, USA; Carl R. Woese Institute for Genomic Biology, University of Illinois Urbana-Champaign, Urbana, IL 61801, USA; Department of Comparative Biosciences, College of Veterinary Medicine, University of Illinois Urbana-Champaign, Urbana, IL 61802, USA; Cancer Center at Illinois, University of Illinois Urbana-Champaign, Urbana, IL 61801, USA; Carl R. Woese Institute for Genomic Biology, University of Illinois Urbana-Champaign, Urbana, IL 61801, USA

## Abstract

The intestinal epithelial regeneration is driven by intestinal stem cells under homeostatic conditions. Differentiated intestinal epithelial cells, such as Paneth cells, are capable of acquiring multipotency and contributing to regeneration upon the loss of intestinal stem cells. Paneth cells also support intestinal stem cell survival and regeneration. We report here that depletion of an RNA-binding protein named polypyrimidine tract binding protein 1 (PTBP1) in mouse intestinal epithelial cells causes intestinal stem cell death and epithelial regeneration failure. Mechanistically, we show that PTBP1 inhibits neuronal-like splicing programs in intestinal crypt cells, which is critical for maintaining intestinal stem cell stemness. This function is achieved at least in part through promoting the non-productive splicing of its paralog PTBP2. Moreover, PTBP1 inhibits the expression of an AKT inhibitor PHLDA3 in Paneth cells and permits AKT activation, which presumably maintains Paneth cell plasticity and function in supporting intestinal stem cell niche. We show that PTBP1 directly binds to a CU-rich region in the 3′ UTR of *Phlda3*, which we demonstrate to be critical for downregulating the mRNA and protein levels of *Phlda3*. Our results thus reveal the multifaceted *in vivo* regulation of intestinal epithelial regeneration by PTBP1 at the post-transcriptional level.

## INTRODUCTION

The intestinal epithelium lines the gastrointestinal tract and plays a critical role in digestion, absorption, and protection against insults from luminal contents. The intestinal epithelium undergoes constant regeneration to maintain its functional integrity physiologically as well as following tissue damage (1,2). Under homeostatic conditions, intestinal regeneration is driven by intestinal stem cells (ISCs) located in the crypt of Lieberkühn. ISCs continuously divide to generate new stem cells and proliferating transit-amplifying cells. Most proliferating transit-amplifying cells migrate up along the crypt-villus axis, during which they differentiate into specialized intestinal epithelial cell (IEC) lineages that execute specific physiological functions. Once the mature IECs reach the tips of villi, they undergo apoptosis and are replaced by new epithelial cells generated by ISCs. A small number of transit-amplifying cells migrate down to the crypt base and differentiate into Paneth cells. Based on the localization and functional properties, two major types of ISC populations have been identified. The first population is the active cycling crypt base columnar (CBC) stem cells which are required for the renewal of IECs under homeostatic conditions (2,3). The second population is the ‘reserve stem cells’ (RSCs) which are activated in response to epithelial damage to replenish the pool of CBC stem cells and repopulate the IECs (2,3). Paneth cells, which are interspersed between CBC stem cells, secrete molecules that support ISC survival and stemness (4–8). Furthermore, Paneth cells are capable of dedifferentiating and acquiring ISC properties to contribute to epithelial regeneration in response to the loss of ISCs (9–12). Despite these important findings, it remains largely unclear how ISC niche and Paneth cell plasticity are precisely maintained.

The phosphoinositide-3-kinase–Akt (PI3K-AKT) pathway is essential for maintaining the survival and regenerative capacity of ISCs under homeostatic conditions and post-injury (13–16). AKT signaling activation is also essential for Paneth cells to acquire ISC properties in response to inflammation (11). AKT is a key effector of the PI3K-AKT pathway. In response to stimulation by a variety of growth factors, AKT is recruited to the cell membrane via interactions between its pleckstrin homology (PH) domain and the membrane lipid phosphatidylinositol-3,4,5-trisphosphate (PIP_3_) (17). The relocation of AKT to the cell membrane allows AKT to be phosphorylated on Thr308 and Ser473 and become activated to induce downstream signaling cascades that inhibit cell apoptosis and promote cell proliferation (17). The activity of PI3K-AKT signaling is antagonized by tumor suppressor gene *p53* in several tissues and cell lines (18–23). One of the P53-mediated AKT inhibitory mechanisms is through the transcriptional activation of AKT repressors. PH-like domain family A, member 3 (PHLDA3), is one such repressor induced by P53, and it possesses a PH domain that allows it to act as a dominant-negative form of AKT (24,25). In cultured cells, PHLDA3 prevents AKT activation by interfering with AKT binding to PIP_3,_ resulting in apoptosis (24,25). While it remains unclear whether PHLDA3 functions *in vivo* to regulate the AKT activity in intestinal regeneration, frequent mutations and copy number variation in the *Phlda3* gene were found in colon carcinoma (https://cancer.sanger.ac.uk/cosmic), which highlights a potential role of PHLDA3 in regulating intestinal homeostasis.

Polypyrimidine tract binding protein 1(PTBP1, also known as HNRNP I) is an RNA-binding protein that serves critical roles in post-transcriptional gene regulation, particularly as a repressor of exon inclusion (26–29). PTBP1-mediated post-transcriptional regulation is essential for embryonic development, cell lineage differentiation, and tissue regeneration (30–44). Among these functions, its role in maintaining the multipotency and self-renewal of stem cells is of particular interest (41–44), which has been most well characterized in the neuronal system. In the brain, PTBP1 is expressed in neuronal progenitor cells to inhibit splicing programs that drive neuronal differentiation (32–34). This inhibitory role of PTBP1 involves repression of its paralog PTBP2 (also called neuronal PTB) that is known to induce neuronal-specific splicing programs (32–34,45–47). How PTBP1 controls stem cell stemness in other tissues remains poorly understood. We previously reported that deletion of the *Ptbp1* gene in mouse neonatal IECs disrupts neonatal immune adaptation and causes early onset of colitis and colorectal cancer (39). Here, we report that in adulthood, PTBP1 controls ISC survival and epithelial regeneration. Mechanistically, PTBP1 inhibits neuronal-like splicing programs in the intestinal crypt cells to keep their stemness, which is achieved at least in part by inhibiting the expression of *Ptbp2*. We further show that PTBP1 inhibits the expression of *Phlda3* in Paneth cells and permits AKT activation, which presumably maintains Paneth cell plasticity and function in supporting the ISC niche. Our results thus reveal a novel mechanism whereby PTBP1 controls intestinal epithelial regeneration through multifaceted post-transcriptional regulations of gene functions.

## MATERIALS AND METHODS

### Animals

 

### Generation of *Ptbp1*^flox/flox^; *Villin-cre*^ERT2/+^ mice

The floxed *Ptbp1* mouse allele *Ptbp1*^flox/flox^ (hereafter *Ptbp1*^f/f^) was generated as previously reported (39). The floxed *Ptbp1* mice were crossed with the *Villin-creERT2* mice (48) to generate the *Ptbp1*^flox/flox^; *Villin-Cre*^ERT2/+^ mice (hereafter *Ptbp1*^f/f^; *Vil-cre*^ER+/−^).

All mice used in this report are from the cross of the *Ptbp1*^f/f^; *Vil-cre*^ER+/−^ mice with the *Ptbp1*^f/f^ mice unless otherwise noted. The tamoxifen administrated *Ptbp1*^f/f^;*Vil-cre*^ER+/−^ mice are referred to as the knockout mice, and their littermates *Ptbp1*^f/f^ mice administrated with the same dose of tamoxifen are referred to as the control mice. Tamoxifen was administrated by single daily injections for two consecutive days at the dose of 100 mg/kg of body weight. Genotyping primers are listed in Supplementary Table S1.

### Generation of *Ptbp1*^flox/flox^;*Vil-cre*^ERT2+/-;^*Lgr5-Egfp-IRES-creERT2* and *Ptbp1*^flox/flox^;*Lgr5-Egfp-IRES-creERT2* mice

*Ptbp1* ^f/f^;*Vil-cre*^ER+/−^ mice were crossed with *Lgr5-Egfp-IRES-creERT2* mice (49) to generate *Ptbp1*^flox/flox^;*Vil-cre*^ERT2+/−^; *Lgr5-Egfp-IRES-creERT2* (hereafter *Ptbp1*^f/f^;*Vil-cre*^ER+/−^;*Lgr5-cre*^ER+/−^) and *Ptbp1*^flox/flox^;*Lgr5-Egfp-IRES-creERT2* (hereafter *Ptbp1*^f/f^; *Lgr5-cre*^ER+/−^) mice. In these mice, *Lgr5*-expression ISCs are labeled with GFP (49) and therefore were used to assess the effect of PTBP1 deficiency on *Lgr5*-expression ISCs. Furthermore, tamoxifen treatment allows deletion of PTBP1 in GFP-positive *Lgr5*-expressing CBC stem cells in *Ptbp1*^f/f^; *Lgr5-cre*^ER+/−^ mice. Genotyping primers used are listed in Supplementary Table S1.

### Ethics statement of animal use

All procedures involving mouse care, euthanasia, and tissue collection have been approved by the University of Illinois Urbana-Champaign Animal Care and Use Committee (IACUC approved protocol #20177 and 20211). Mice were used and cared according to the institutional ‘Guide for the Care and Use of Laboratory Animals’ and in accordance with all University of Illinois Urbana-Champaign policies and guidelines outlining the care and use of animals in research.

### Weight loss measurement and death record

Mouse body weight was measured before tamoxifen injection and every 24 h after tamoxifen injection for 5 consecutive days. Percent weight loss was calculated by subtracting the weight measured at 24, 48, 72 h, etc., post tamoxifen injection from the weight measured at 0-hour post tamoxifen injection and dividing by 0-hour weight. Mice were monitored every day after tamoxifen injection for at least two weeks.

### Histology and immunostaining

Intestines were isolated, fixed in 4% paraformaldehyde at 4°C overnight, paraffin-embedded, and sectioned according to the standard protocols. Intestine sections (5 μm) were processed for hematoxylin and eosin staining or for immunostaining. Immunohistochemistry was performed using the R.T.U. vectastain kit (Vector Laboratories) with DAB substrate and sections were counterstained lightly with hematoxylin afterward. Double immunofluorescence staining with two antibodies produced in rabbits was done by following a protocol described at the Jackson Immune Research Laboratory website (https://www.jacksonimmuno.com). Briefly, after the first secondary antibody incubation, rabbit serum was applied to saturate open binding sites on the first secondary antibody. This was followed by applying excessive unconjugated fab goat anti-rabbit IgG (H + L) fragments on the sections to cover the rabbit IgG to prevent the binding of the second secondary antibody to the first primary antibody. Images were taken from a Leica compound microscope with a digital camera or a Nikon A1R confocal microscope and processed using Adobe Photoshop.

### Antibodies

Secondary antibodies used are goat anti-rabbit or mouse AlexaFluor 488 or 594, donkey anti-goat or rabbit AlexaFluor 488 or 594 (all from Invitrogen). Sections were counterstained with 4′,6-diamidino-2-phenylindole (DAPI) (Sigma, D8417). Primary antibodies used are rabbit anti-active CASPASE 3 (Cell Signaling, 9661), mouse anti-KI67 (BD Pharmingen, 550609), rabbit anti-OLFM4 (Cell Signaling, 39141), goat anti-PTBP1 (Santa Cruz sc-16547), rabbit anti-PTBP1 (gift from Dr. Douglas Black), goat anti-GFP (Rockland, 600-101-215), rabbit anti-P53 (Leica Biosystems, NCL-L-p53-CM5p), rabbit anti-PHLDA3 (LifeSpan BioSciences, LS-C499531-100), rabbit anti-Lysozyme antibody (Diagnostic Biosystems, RP028), and Affinipure Fab Fragment Goat anti-Rabbit IgG (H + L) (Jackson ImmunoResearch, 111-007-003).

### Intestinal organoid culture and whole mount immunostaining

Crypts of the small intestine were isolated from the *Ptbp1*^f/f^; *Vil-cre*^ER+/−^ and *Ptbp1*^f/f^ mice as previously described (50). Briefly, the small intestines were dissected and washed with cold phosphate buffered saline (PBS). After surface villi were scraped off, intestinal segments were cut into 0.5 cm pieces and incubated in 2 mM EDTA/PBS for 30 min. After washing with PBS, intestinal pieces were shaken vigorously to release crypts, and the supernatants were filtered through 70 μm filters to collect the filtrate. The crypt release and filtering steps were repeated 2 times to collect 3 fractions. The fractions that were enriched with crypts were centrifuged at 200 g at 4°C for 5 min to separate crypts from single cells. The supernatants were discarded, and the crypt pellets were resuspended in 5 mL cold DMEM/F-12 and used for organoid culture. Organoids were cultured in IntestiCult Organoid Growth Medium (Stem Cell Technology, 06005). 4-hydroxytamoxifen was added into the culture medium at a final concentration of 200 nM to delete *Ptbp1*. After 24 h of incubation, 4-hydroxytamoxifen was removed by replacing it with a fresh medium. The culture medium was changed every 48 h.

For whole mount immunostaining, organoids were fixed in 4% paraformaldehyde at 4°C overnight and permeabilized with 0.5% Triton X-100 in PBS. Organoids were then treated with 100 mM glycine in PBS to block free aldehyde groups. After being washed with PBS, organoids were treated with a blocking buffer containing 5% serum for 90 min and incubated with the primary antibody overnight at 4°C. After washing with PBS, organoids were incubated with the secondary antibody in the dark at room temperature for 2 h and counterstained with DAPI. Images were taken from a Nikon A1R confocal microscope and processed using Adobe Photoshop.

### Quantitative real-time PCR

Small intestinal crypt cells were isolated as described above at 20, 24, 36, 48, and 50 h post tamoxifen induction. RNAs were extracted using TRIzol reagent according to standard protocols. Real-time PCR reactions were performed blindly in triplicate or duplicate using SYBR green master mix. PCR primers used are listed in Supplementary Table S2.

### Western blots

Isolated small intestinal crypt cells were homogenized in the lysis buffer as previously described (39). Protein lysates were cleared by spinning the samples twice at 4°C. Subsequently, samples were separated on SDS-PAGE and analyzed by western blotting as previously described (39). Primary antibodies used are mouse anti-PTBP1 (Life Technologies, 324800), mouse anti-PTBP2 antibody (ABCAM, sc-376316), rabbit anti-Pan AKT (Cell Signaling, 4685), rabbit anti-Phospho-AKT (Ser473) (Cell Signaling, 4060), mouse anti-HSC70 (Santa Cruz Biotechnology, SC-7298), and rabbit anti-GRP78 (Abcam, ab32618). Membranes were incubated with HRP-linked secondary antibodies and developed using ECL prime (G&E Healthcare Life Sciences).

### *Phlda3* reporter constructs and *in vitro* transcription

GFP-myc-SV40 was described previously (51). GFP-myc-mPhlda3 3′ UTR constructs and pCS2-myc-mPTBP1 were generated by standard PCR cloning methods. All mRNAs used in this study were synthesized from 2 μg of plasmid templates using the mMESSAGE mMACHINE SP6 Transcription kit (Ambion, AM1340).

### *Phlda3 in vitro* binding assay

To obtain myc-mPTBP1 protein, *in vitro* translation was performed using the rabbit reticulocyte lysate system (Promega, L4960) according to the manufacturer's instructions. After translation, MYC antibodies (Invitrogen, 13-2500) and Dynabeads Protein G (Thermo Fisher Scientific, 10004D) were added into the *in vitro* translation reaction and incubated at 4°C overnight. Beads were washed extensively with RIPA buffer. The protocol for *in vitro* RNA pulldown assay was described previously (52). Briefly, myc-mPTBP1-bound Dynabeads Protein G were incubated with 10 μg yeast tRNA in 1 ml RIP buffer (50 mM Tris pH 7.6, 125 mM NaCI, 1 mM EDTA, 0.25% NP-40, 0.2% glycerol, 0.1 mM dithiothreitol) at 4°C for 1 h. Subsequently, 100 ng of each synthesized *Phlda3* 3′ UTR RNAs were added and incubated at 4°C for 4 h. Afterward, beads were washed with RIP buffer five times. RNAs associated with the beads were recovered using TRIzol reagent for RT-qPCR.

### Cell culturing and transfection of *Phlda3* reporter constructs

SW480 cells were cultured in a complete cell culture medium (RPMI1640) (Thermo Fisher Scientific, 22400-089) supplemented with 10% fetal bovine serum, 1% penicillin-streptomycin at 37°C in a humidified incubator supplied with 5% CO2. Transfection was performed using PEI, and 0.5 ug of each plasmid was used for the experiment. The plasmids were mixed with PEI, and incubated in OptiMEM (Thermo Fisher Scientific, 31985-070) for 20 min at room temperature. Afterward, the complex was transfected into SW480 cells and incubated for 4 h. Then, OptiMEM containing plasmid/PEI complexes were discarded, replaced with a complete cell culture medium, and incubated for 24 h. After transfection, cells were collected for further analysis.

### RNA extraction, RT-qPCR and western blot for *Phlda3* reporter constructs

RNAs were extracted from transfected SW480 cells using TRIzol reagent. cDNA synthesis and qPCR were performed using the M-MLV Reverse Transcriptase (Promega, M1701) and Bimake 2× SYBR Green qPCR Master Mix (Bimake, B21203), respectively. PCR primers used are listed in Supplementary Table S2. Ct values were acquired using Applied Biosystems QuantStudio 3 Real-Time PCR System. For western blots, cells were lysed using lysis buffer (50 mM Tris pH 7.6, 125 mM NaCI, 1 mM EDTA, 1% NP-40), mixed with 2× SDS sample buffer, and boiled for 10 min at 100°C. The rest of the procedure was followed by standard western blotting protocol. Goat anti-GFP (Rockland, 600-101-215) and mouse anti-HSC70 (Santa Cruz, sc-7298) were used as primary antibodies.

### RNA-seq analysis

Crypt cells from small intestines were isolated at 20 h post tamoxifen administration from 3 knockout mice and 3 littermate control mice. RNAs were extracted using PureLink RNA Mini Kit (Ambion, Cat. 12183025). RNA-seq libraries were constructed and sequenced at the University of Illinois Urbana-Champaign Biotechnology Center High-Throughput Sequencing Core. Sequencing was done by using 150 base pairs of paired-end reads. Each library generated over 140 million paired reads. Raw reads were subjected to read length and quality filtering using Trimmomatic V0.38 (53) and aligned to the mouse genome (mm10) using STAR (version 2.6.1d) (54). Cufflinks package (55) was used to assess differential gene expression events, among which significant events were identified using a stringent cutoff criteria: FDR(q-value) < 0.05, FPKM ≥ 1 and log_2_(fold change) ≥1. rMATS v4.0.2(turbo) (56) was used to study differential splicing, and events with FDR <0.1, junction read counts ≥10, and PSI ≥10% were deemed to be significant.

Exon ontology analysis was performed on the set of alternatively spliced cassette exons identified using rMATS. Mouse(mm10) annotations were converted to human (hg19) annotations using UCSC liftover with a minimum base remap ratio set to 0.8. Exon ontology pipeline (57) was then used on the lifted exons to perform ontology analysis.

### Motif analysis

K-mer enrichment and de-novo motif analysis were performed using the set of regulated cassette exon sequences and/or intronic region 100 bp upstream/downstream to them. For each analysis, corresponding sequences from the set of all cassette exons and their proximal regions were used as background. kpLogo was used to generate k-mer logos from upstream and downstream intronic regions (58). De-novo motif discovery was performed using MEME in Differential Enrichment mode considering Any Number of Repetitions (anr) for motifs (59). A motif enrichment map for alternatively spliced exons was constructed using RMAPs with a 50-nucleotide sliding window (60).

### PCR-based splicing assay

PCR-based splicing assays were performed by using primers that target the constitutive exons flanking the alternative spliced exons. The products were resolved on a 5% poly-acrylamide gel and imaged using ethidium bromide staining on a Biorad ChemiDoc XRS+ imaging system. Quantification of gel images was done using Image Lab 5.2.1 software (Biorad). PSI values were determined as [the exon inclusion band intensity/(the exon inclusion band intensity + the exon exclusion band intensity)] × 100. PCR primers used are listed in Supplementary Table S3.

### Crosslinked RIP-qPCR

Crosslinked RIP-qPCR was performed from SW480 cells using an adapted eCLIP protocol (61). Briefly, cells were subjected to UV crosslinking (254 nm, 400 mJ/cm^2^), lysed in 1ml iCLIP lysis buffer, and digested with Turbo DNase (10 min at 37°C). PTBP1 was pulled down from crosslinked lysate using 3 ug of anti-PTBP1 antibody (clone BB7, MABE986) conjugated to anti-mouse Dyna beads, washed with wash buffer, and subjected to proteinase-K digestion. RNA was extracted using acid phenol/chloroform/isoamyl alcohol (pH 6.5), reverse-transcribed using Maxima H reverse transcriptase, quantified using qPCRs (*PTBP2_RIP_Fp* and *PTBP2_RIP_Rp*) and normalized to respective inputs. PCR primers used are listed in Supplementary Table S4.

### Statistical analyses

Differences between the knockout mice and the control groups were assessed for significance using a two-tailed unpaired Student *t*-test unless otherwise noted. Data involving two or more variables were analyzed by two-way ANOVA using GraphPad Prism.

## RESULTS

### IEC-specific *Ptbp1* deletion in adulthood results in impaired intestinal epithelium regeneration

**Figure 1.**
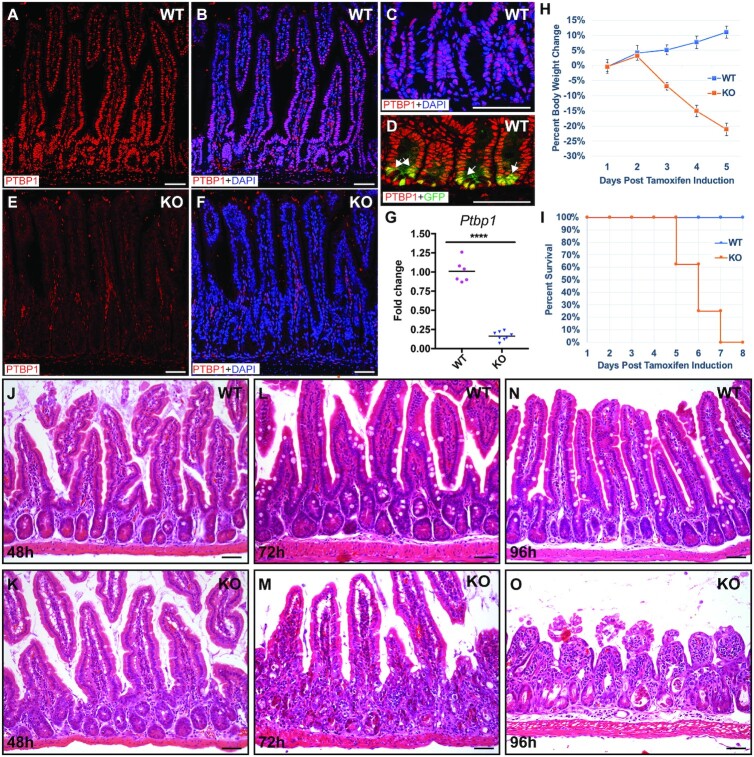
IEC-specific *Ptbp1* deletion results in failure of intestinal epithelium regeneration. (**A**–**F**) Immunofluorescence staining using an anti-PTBP1 antibody shows PTBP1 protein localization in the wild-type and *Ptbp1* knockout small intestine. Nuclear accumulation of PTBP1 protein was detected in all IECs, including LGR5-positive CBC stem cells in wild-type mice (arrows in D). LGR5-positive CBC stem cells were detected by an anti-GFP antibody on intestinal tissues of non-tamoxifen treated *Ptbp1*^f/f^;*Lgr5*^ER+/−^ mice. In the knockout mice, PTBP1 expression is diminished in the IECs but remains unaffected in the lamina propria cells at 48 h PTI (E and F). Nuclei were counterstained with DAPI. (**G**) Quantitative Real-time PCR shows a significantly decreased level of *Ptbp1*mRNA in the intestinal crypt cells of the knockout mice at 48 h PTI. Data were normalized to *Gapdh*. Each symbol in the graph indicates fold change of *Ptbp1* expression in individual mice. Bars show mean values. Mice of the knockout group and the wild-type group are sibling littermates. The data shown are representative of at least three independent experiments. (**H**) shows a weight loss of *Ptbp1* knockout mice within 5 days PTI. (**I**) shows the death of *Ptbp1* knockout mice within 8 days PTI. Data in H and I were from N = 8 knockout mice and 9 control mice. (**J–O**) Hematoxylin and eosin–stained sections show changes in the epithelial structures of the control mice and *Ptbp1* knockout mice at 48 h (J and K), 72 h (L and M), and 96 h (N and O) PTI. WT, wild-type; KO, knockout. Scale bars, 50 μm. **** *P* < 0.0001.

Previously, we investigated the function of PTBP1 in the intestinal epithelium by deleting the *Ptbp1* gene in IECs from early embryogenesis using *Villin-Cre* (39). While this mouse model allowed us to gain valuable insights into the role of PTBP1 in neonatal IECs, it is not applicable to studying the function of PTBP1 in the adult intestinal epithelium. Therefore, we generated a tamoxifen-inducible *Ptbp1* knockout mouse model *Ptbp1*^f/f^; *Vil-cre*^ER+/−^ mice. We found that PTBP1 protein is highly accumulated in the nuclei of all IECs, including *Lgr5*-expressing CBC stem cells in the wild-type mice (Figure 1A–D). Tamoxifen treatment induced a near-complete depletion of PTBP1 in the entire IECs of *Ptbp1*^f/f^; *Vil-cre*^ER+/−^ mice, as indicated by nondetectable PTBP1 protein in the IECs and a significant decrease in the level of *Ptbp1* mRNA at 48 h post tamoxifen induction (PTI) (Figure 1E–G).

Strikingly, all *Ptbp1* knockout mice showed significant weight loss and died within 7 days PTI (Figure 1H and I). To determine the cause of death, we collected intestine tissues every 24 h PTI and performed histological analysis. We found that the overall organization of the intestinal epithelium in the knockout mice appeared relatively normal at 48 h PTI (compare Figure 1J to K). By 72 h PTI, knockout mice displayed destruction of the epithelial structure due to the detachment of the intestinal epithelial layer from lamina propria and crypt cell death (compare Figure 1L to M). The regular crypt-villus architecture was completely disrupted in the knockout mice at 96 h PTI, displaying villous atrophy (Compare Figure 1N to O). This finding indicates that loss of PTBP1 results in failure of intestinal epithelial regeneration.

### Loss of ISCs in *Ptbp1* knockout mice

**Figure 2.**
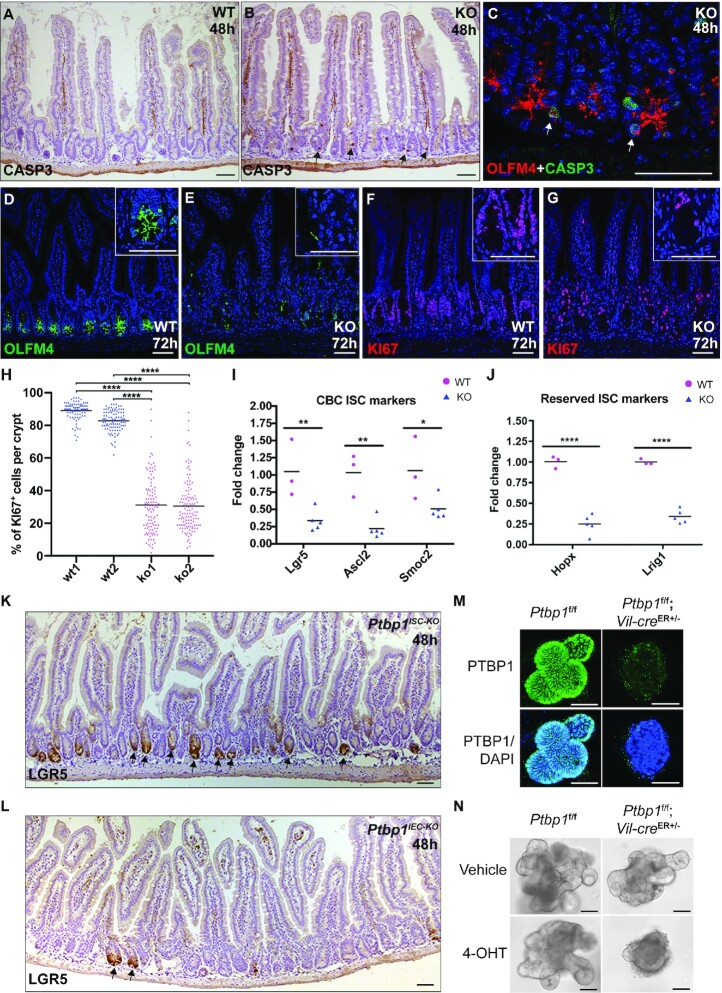
IEC-specific PTBP1 deficiency results in the loss of intestinal stem cells. (**A**, **B**) Immunohistochemical staining with an anti-cleaved CASPASE 3 antibody shows an increased number of apoptotic cells in the crypt region of knockout mice at 48 h PTI (B, arrows). (**C**) Double immunofluorescence staining with anti-OLFM4 and anti-cleaved CASPASE 3 antibodies shows the death of OLFM4-positive stem cells (arrows in C). (**D–G**) Immunofluorescence staining shows knockout mice have a significant decrease in the number of OLFM4-positive cells (E) and KI67-positive cells (G) at 72 h PTI. (**H**) showing the quantification of KI67-positive cells in 2 knockout mice and 2 control sibling mice. Each symbol in the graph indicates the percentage of KI67-positive cells in individual crypts. Bars show mean values. (**I-J**) Real-time PCR shows decreased expression of CBC stem cell markers (**I**) and RSC markers (J). Data were normalized to *Gapdh*. Each symbol in the graphs indicates the fold change of gene expression in individual mice. Bars show mean values. Mice of the knockout group and the wild-type group are sibling littermates. All data shown are representative of at least three independent experiments. (**K-L**) Immunohistochemical staining with an anti-GFP antibody shows LGR5-positive CBC stem cells in the intestines of tamoxifen-administrated *Ptbp1*^f/f^;*Vil-cre*^ER+/−^;*Lgr5*^ER+/−^ mice (denoted by *Ptbp1*^IEC-KO^) and *Ptbp1*^f/f^;*Lgr5*^ER+/−^ mice (denoted by *Ptbp1*^ISC-KO^) at 48 h PTI. Arrows point to LGR5-positive crypts. (**M**) Whole mount immunofluorescence staining with an anti-PTBP1 antibody shows efficient depletion of PTBP1 in 4-OHT-treated organoids derived from *Ptbp1*^f^*^/^*^f^;*Vil-cre*^ER+/−^ mice but not those from *Ptbp1*^f/f^ mice at day 3 of culture. Organoids were counterstained with DAPI. (**N**) 4-OHT-treated organoids derived from *Ptbp1*^f^*^/^*^f^;*Vil-cre*^ER+/−^ mice failed to bud at day 4 of culture, but not those from the control mice. Vehicle-treated organoids from *Ptbp1*^f^*^/^*^f^;*Vil-cre*^ER+/−^ mice and the control mice displayed normal budding. WT, wild-type; KO, knockout. 4-OHT, hydroxytamoxifen. Scale bars, 50 μm. * *P* < 0.05; ** *P* < 0.01; **** *P* < 0.0001.

To determine if villous atrophy in the knockout mice is caused by loss of ISCs, we first assessed cell apoptosis in the crypt region of the knockout mice and detected a dramatic increase in the number of cleaved CASPASE3-positive cells at 48 h PTI (compare Figure 2B to A, Table 1, Supplementary Figure S1). While crypt cell death was observed in both the small and large intestines of the knockout mice, the small intestine was more severely affected by PTBP1 deletion, as indicated by the percentage of cleaved CASPASE3-positive crypts in the small and large intestines (Table 1). We, therefore, focused our studies on the small intestine.

**Table 1.** Quantification of crypt cell death at 48 h post tamoxifen injection

Genotype	Control mice small intestine	KO mice small intestine	Control mice colon	KO mice colon
Number of crypts examined	605	728	665	573
Percentage of cleaved CASPASE3-positive crypts	0.3%	43.7%	0.6%	9.9%

We next checked if ISCs undergo apoptosis in the knockout mice by assessing the colocalization of cleaved CASPASE3 and OLFM4, a CBC stem cell marker (62). Indeed, we detected cleaved CASPASE3 staining in OLFM4-positive ISCs at 48 h PTI (Figure 2C), demonstrating apoptosis of ISCs in the knockout mice. Consistent with this finding, the expression of OLFM4 was diminished in the crypt cells of the knockout mice at 72 h PTI (compare Figure 2D to E). This was accompanied by a significant reduction in the number of proliferating crypt cells at 72 h PTI in the knockout mice (Figure 2F–H). To determine whether both CBC stem cells and RSCs are affected, we assessed the expression level of genes specific to CBC stem cells or RSCs. The expression levels of *Lgr5*, *Ascl2* and *Smoc2* (signature genes for CBC stem cells) and *Hopx* and *Lrig1* (RSC markers) were significantly reduced in the knockout mice (Figure 2I and J), indicating loss of both ISC populations. Collectively, these results demonstrate that the epithelial PTBP1 plays a critical role in maintaining the survival and proliferation of ISCs.

To understand if loss of PTBP1 in ISCs alone is sufficient to induce ISC apoptosis, we compared the number of *Lgr5*-expresing ISCs in tamoxifen-administrated *Ptbp1*^f/f^;*Lgr5*^ER+/−^ mice and *Ptbp1*^f/f^;*Vil-cre*^ER+/−^;*Lgr5*^ER+/−^ mice. In these mice, LGR5-positive CBC stem cells are labeled with GFP due to variegated expression of the *Lgr5-Egfp-IRES-CreERT2* transgene (49). Since the CreERT2 fusion protein is expressed in GFP-positive *Lgr5*-exressing ISCs (49), we were able to delete PTBP1 specifically in GFP-expressing ISCs in *Ptbp1*^f/f^; *Lgr5*^ER+/−^ mice upon tamoxifen treatment (Supplementary Figure S2A–D). Our results show that deletion of PTBP1 in the *Lgr5*-expressing ISCs did not eliminate these cells at 48 h PTI (Figure 2K, Table 2), and the intestinal epithelial structure appeared normal at 72 PTI (Supplementary Figure S2E). On the contrary, deletion of PTBP1 in the entire IECs by tamoxifen administration to the *Ptbp1*^f/f^;*Vil-cre*^ER+/−^;*Lgr5*^ER+/−^ mice resulted in a significant reduction in the number of the GFP-positive *Lgr5*-expressing ISCs at 48 h PTI (Figure 2L, Table 2). This was accompanied by the destruction of the epithelial structure at 72 h PTI (Supplementary Figure S2F). This finding suggests that loss of PTBP1 in *Lgr5*-expressing ISCs alone is not sufficient to provoke the death of these cells, and PTBP1 function in other IEC linages is critical for ISC survival, presumably by providing an ISC niche.

**Table 2.** Quantification of crypts that contain LGR5-expressing CBC stem cells at 48 h PTI based on the GFP expression

Genotype	*Ptbp1* ^f/f^; *Lgr5*^ER+/−^	*Ptbp1* ^f/f^; *Vil-cre*^ER+/−^; *Lgr5*^ER+/-^ mouse 1	*Ptbp1* ^f/f^; *Vil-cre*^ER+/−^; *Lgr5*^ER+/−^ mouse 2
Number of crypts examined	848	843	783
Percentage of crypts that contain LGR5-expressing cells	38.0%	8.9%	9.7%

To further determine if PTBP1 is required for maintaining the ISC regenerative capacity, we established an *ex vivo* intestinal organoid culture system using crypts isolated from *Ptbp1*^f/f^; *Vil-cre*^ER+/−^ mice and their control littermate *Ptbp1*^f/f^ mice. Efficient deletion of *Ptbp1* was observed in *Ptbp1*^f/f^; *Vil-cre*^ER+/−^ organoids upon 4-hydroxytamoxifen treatment for 24 h (Figure 2M). We found that 4-hydroxytamoxifen-treated *Ptbp1*^f/f^; *Vil-cre*^ER+/−^ crypts failed to bud at day 4 of culture when compared to vehicle-treated *Ptbp1*^f/f^; *Vil-cre*^ER+/−^ crypts or 4-hydroxytamoxifen-treated *Ptbp1*^f/f^ crypts (Figure 2N). This observation further demonstrates that PTBP1 is required for ISC-mediated epithelial regeneration.

### Transcriptome changes in PTBP1-deficient cells

**Figure 3.**
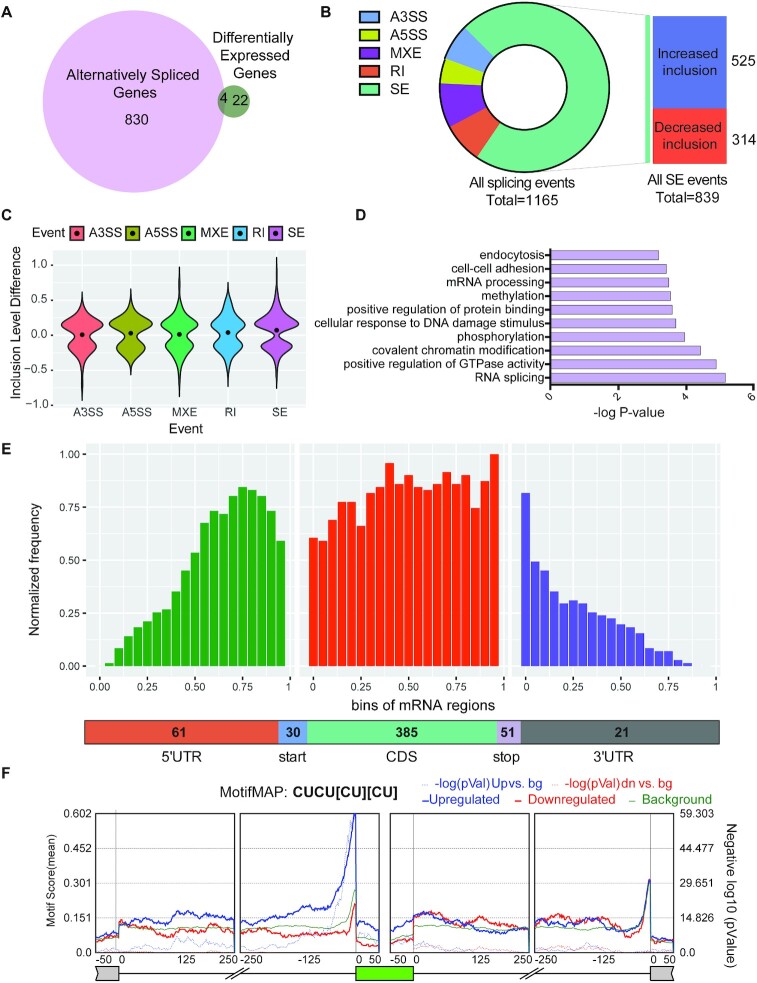
Transcriptome changes in PTBP1-deficient crypt cells. (**A**) Venn diagram showing the number of genes in which differential gene expression/alternative splicing changes were detected in the knockout crypt cells. Four genes (*Trim72*, *Ager*, *H2-Bl*, and *Fmr1nb*) displayed changes in both gene expression and splicing. (**B**) Breakdown of 1165 alternatively spliced events into various event categories. A3SS: Alternative 3′ splice site, A5SS: Alternative 5′ splice site, MXE: Mutually exclusive exon, RI: Retained intron, SE: Skipped exon. (**C**) Violin plots demonstrating ΔPSI (Inclusion level difference) distributions of significantly altered splicing events in the knockout crypt cells. (**D**) Gene ontology analysis demonstrates the top 10 biological processes that are enriched among genes that are alternatively spliced. (**E**) Metagene analysis of alternatively spliced exons in knockout mice by their positions on mRNA transcripts. Distribution along the transcript bins is shown on top, and the breakup of events into relative transcript regions is shown at the bottom. (**F**) shows the relative enrichment of [CT]-rich motif near cassette exons (represented in green) that displayed a significant increase (blue curve) or decrease (red curve) in inclusion in PTBP1-deficient crypt cells. Alternatively spliced exons were identified using rMATS, and a motif map was constructed using RMAPs (with a 50-nucleotide sliding window). The set of background cassette exons is represented in black.

To determine the molecular mechanism by which PTBP1 controls the survival and regeneration of intestinal crypt cells, we deep-sequenced poly(A) selected RNAs prepared freshly from age-matched wildtype and PTBP1-deficient crypt cells. We chose to assess the transcriptome changes in the knockout mice at 20 h PTI, a time point before cell death could be detected in the crypt region so that the observed changes represent the direct consequence of PTBP1 deficiency and are likely the cause of the crypt cell death. Our results show that PTBP1 deletion predominantly affects mRNA splicing (Figure 3A). We identified a total of 1165 altered splicing events within 834 genes (Figure 3A and B, Supplementary Table S5). Remarkably, in contrast to a large number of splicing changes, only 26 genes showed changes in their mRNA abundance at 20 h PTI (Figure 3A and Supplementary Table S6). Amongst them, 4 genes (*Trim72*, *Ager*, *H2-Bl*, and *Fmr1nb*) exhibited significant differences in both mRNA abundance and splicing. These findings provide strong evidence that the splicing defects triggered by PTBP1 deficiency are primary events that precede the onset of crypt cell death in the PTBP1 knockout intestines.

The majority of splicing changes in PTBP1-deficient crypt cells were exon skipping events (72%), but changes in alternative 5′ or 3′ splice sites, intron retention, and mutually exclusive exons were also detected (Figure 3B). Notably, nearly two-thirds of skipped exons displayed increased inclusion in PTBP1-deficient crypt cells (Figure 3C), which is consistent with the previous reports that PTBP1 primarily functions as a repressor of splicing (29). Gene ontology analysis further revealed that the differentially spliced mRNAs following *Ptbp1* deletion were enriched in several functional clusters, including mRNA splicing (Supplementary Table S7), regulation of GTPase activity, chromatin modification, phosphorylation, and cellular response to DNA damage stimulus (Figure 3D).

We next investigated the spatial distribution of PTBP1-regulated exons in the crypt cells along their associated transcripts, as previously described (63). Metagene analysis revealed that approximately 70% of differentially spliced exons were located within coding sequences (CDS), and a sizeable number of those (15%) encoded alternate START or STOP codons (Figure 3E). We then performed *de novo* motif discovery (for 4–8 bp long motifs) and position-specific k-mer enrichment (for 6-mers) in the regulated cassette exons and/or proximal intronic region. We observed a significant enrichment of the YCUY motif and CU-rich 6-mers in the upstream intronic region surrounding the cassette exons that were abnormally included in PTBP1-deficient crypt cells (Supplementary Figures S3 and S4). Furthermore, we found a strong overrepresentation of the CUCUCUCU motif near the 3′ splice site of exons that are more included upon PTBP1 depletion. (Figure 3F). These CU/pyrimidine-rich motifs represent direct binding motifs for PTBP1 (64–66), suggesting that in crypt cells, PTBP1 suppresses the inclusion of many alternate exons by directly binding to its motif in the upstream introns.

**Figure 4.**
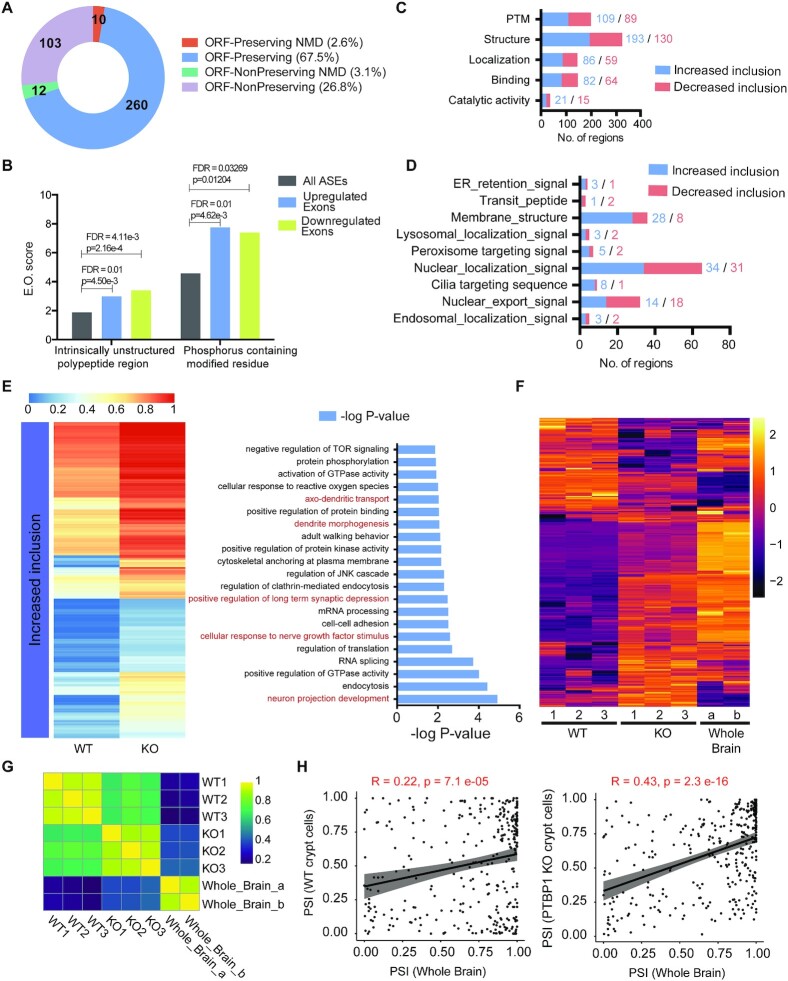
Detailed analysis of exons misregulated in the skipped exon category. (**A**) Effect of alternatively spliced events induced by PTBP1 deficiency on the transcripts in crypt cells. Categories indicate if the exon inclusion preserves the open reading frame (ORF) and if the inclusion transcript is subjected to Non-sense Mediated Decay (NMD). (**B**) Relative to a set of all Alternatively Spliced Exons (ASEs), PTBP1-regulated exons (either upregulated or downregulated) are significantly enriched for regions encoding intrinsically unstructured regions and phosphorylation sites. Exon Ontology (E.O.) scores are shown on the y-axis. (**C**) Exon ontology-based distribution of skipped exons, either increase or decrease in inclusion, based on encoded protein features. PTM: post-translational modification. (**D**) Exon ontology-based distribution of differentially skipped exons based on localization. (**E**) The heatmap shows the average Percentage Spliced In (PSI) values of exons that have significantly increased inclusion in the knockout mice. Corresponding gene ontology analysis reveals the biological processes that are enriched among these genes (shown on the right). Neuronal cell differentiation-related biological processes are highlighted in red. (**F**) Heatmap shows PSI values of significantly altered splicing events between WT and PTBP1-deficient crypt cells compared to PSI values from the whole brain (obtained from VastDB database). (**G**) The plot demonstrates Pearson's correlation of PSI values obtained from WT, KO, and whole brain datasets. Compared to wt, the ko dataset displays a higher correlation with the whole brain. (**H**) PSI scatter plots that compare wt or ko datasets with whole brain datasets for evaluation of Pearson's correlation.

The CDS-mapped PTBP1-regulated exons were further classified based on whether they were open reading frame preserving (exon length is a multiple of 3). We found that 70% of the regulated exons preserve the open reading frame, whereas 30% of them do not (Figure 4A). In both cases, only about 3% of the exons were predicted to undergo nonsense-mediated RNA decay (Figure 4A). This result is consistent with our transcriptome data wherein most of the mRNAs harboring PTBP1-regulated exons in crypt cells do not exhibit a significant change in their overall abundance (Figure 3A). On the contrary, these exons are likely to alter the intrinsic structure and function of the encoded proteins. To further probe the functional properties and features of PTBP1-regulated exons, we performed exon ontology analysis, which revealed significant enrichment for sequences encoding intrinsically unstructured regions, phosphorylation sites, post-translational modifications, cellular localization, binding, and catalytic activity (Figure 4B and C). We further noted that within the cellular localization category, many PTBP1-regulated exons contained a nuclear localization, export, or membrane targeting signal (Figure 4D).

Gene ontology analysis revealed that the differentially spliced genes due to increased exon inclusion in PTBP1-deficient crypt cells function in a number of biological processes related to neuronal cell differentiation, including neuron projection development, cellular responses to nerve growth factor stimulus, regulation of synaptic depression, dendrite morphogenesis, and axo-dendritic transport (Figure 4E, detailed in Supplementary Table S8). Using RT-PCR, we validated the increase in exon inclusion in several neuronal genes in PTBP1-deficient crypt cells (Supplementary Figure S5A and B). This observation prompted us to investigate whether loss of PTBP1 in crypt cells induces global neuronal-like alternative splicing patterns as seen in the brain. We compared the PSI values seen in the control and knockout crypt cell datasets to the wild-type brain dataset obtained from the publicly available well-compiled alternative splicing database (VastDB) (67,68). We found that, on average, the differentially spliced events in PTBP1-deficient crypt cells displayed inclusion levels closer to those in the brain when compared to the control (Figure 4F to H), indicating a shift toward a neuronal-like splicing pattern. These findings suggest that PTBP1 represses splicing programs required for neuronal differentiation in ISCs and transit-amplifying cells, which is presumably important for maintaining the multipotency of these cells. Interestingly, we found that the expression levels of key transcriptional factors that promote neuronal differentiation were not affected by the loss of PTBP1 (Supplementary Figure S5C), suggesting that PTBP1 predominately inhibits neuronal-like splicing patterns in crypt cells and is critical for maintaining ISC stemness, but its loss is not sufficient to induce successful neuronal differentiation in the crypt cells. In addition to neuronal-like splicing, we validated 11 exon-skipping events and observed an excellent correlation of experimentally calculated ΔPSI values with ΔPSI values determined in our computational analysis (Supplementary Figure S6).

### PTBP1 suppresses PTBP2 expression at the mRNA and protein levels in crypt cells

**Figure 5.**
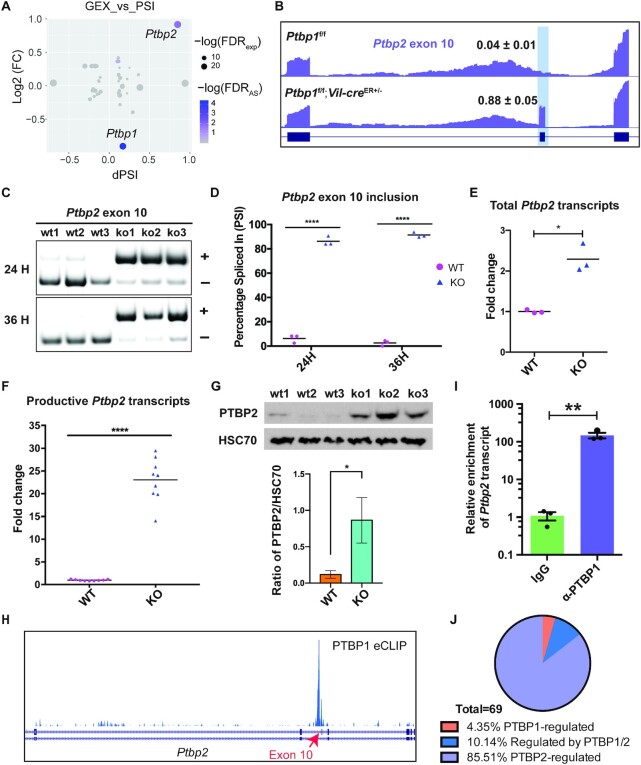
PTBP1 inhibits *Ptbp2* expression through repressing *Ptbp2* exon 10 inclusion. (**A**) Scatter plot demonstrates significant alternative-splicing changes among RBPs observed in PTBP1 KO IECs (x-axis), and changes in expression levels of the corresponding RBP (y-axis). Each dot represents a splicing event in an RBP, and its color and size represent negative logarithmic FDR values of splicing change in a particular RBP transcript exon and changes in expression levels of the corresponding RBP, respectively. (**B**) UCSC Track image shows high inclusion of exon 10 of *Ptbp2* in the knockout crypt cells. (**C**, **D**) PCR-based splicing assay on exon 10 inclusion in *Ptbp2* mRNA at 24 and 36 h PTI. Gel images show a significant increase in exon 10 inclusion (C). Exon inclusion and exclusion bands are denoted by (+) and (–), respectively. Bars show mean values (D). WT and KO mice used for analysis are littermates. (**E**, **F**) Real-time PCR shows the upregulation in the levels of total *Ptbp2* transcripts (E) and productive *Ptbp2* transcripts at 20 h PTI in PTBP1 KO crypt cells. Data were normalized to *Gapdh* and each dot in the graphs indicates a fold change of *Ptbp2* expression in individual mice. Bars show mean values for each group. Primers used for detecting the total *Ptbp2* transcripts target the constitutive exons of *Ptbp2*. The reverse primer used for detecting the productive *Ptbp2* transcripts targets the exon 10 sequence of *Ptbp2*. (**G**) Western blot shows PTBP2 protein expression is significantly increased in the knockout crypt cells at 24 h PTI. HSC 70 is served as the loading control. Quantification of PTBP2 protein level is shown in the low panel. (**H**) UCSC track visualization of PTBP1 eCLIP data from HepG2 cells shows PTBP1 binding site on the *Ptbp2* transcript. (**I**) Crosslinked RIP-qPCR results demonstrate direct binding of PTBP1 to the *Ptbp2* transcript. *Ptbp2* is significantly enriched in pulldown using an anti-PTBP1 antibody when compared to the one using IgG (*t*-test, *P*= 0.004). All samples were normalized to respective input controls. (**J**) A pie chart shows alternative splicing events in PTBP1-deficient crypt cells that are reported to be PTBP1 or PTBP2 regulated in the mouse neocortex. The classifications were made based on published PTBP1 or PTBP2 specific alternative splicing regulation profile (33). * *P* < 0.05; ** *P* < 0.01; **** *P* < 0.0001. WT, wild-type; KO, knockout.

In the brain, PTBP1 suppresses neuronal-specific splicing programs in neuronal progenitor cells through inhibiting *Ptbp2* expression (33,34,45,46,69,70). This is by repressing the inclusion of the alternative exon 10 in the *Ptbp2* transcript, which leads to nonsense-mediated RNA decay of *Ptbp2* (32,34,45). To determine if a similar repression mechanism exists in the crypt cells, we assessed *Ptbp2* splicing and expression. We found that among all RNA-binding proteins that showed altered splicing or expression upon loss of PTBP1, PTBP2 is the most affected one (Figure 5A). We observed a striking increase in the inclusion of *Ptbp2* exon 10 in PTBP1-deficient crypt cells at 24 and 36 h PTI (Figure 5B–D). This was accompanied by approximately a net two-fold increase in the levels of total *Ptbp2* transcripts (Figure 5E, Supplementary Table S9) and a much significant upregulation in the levels of *Ptbp2* transcripts that contains exon 10 (Figure 5F). Because *Ptbp2* transcripts containing exon 10 are productive and are not subjected to NMD, PTBP2 protein levels are dramatically upregulated as well (Figure 5G). To investigate if PTBP1 directly represses *Ptbp2* exon 10 inclusion, we analyzed the publicly available HepG2 PTBP1 eCLIP data from ENCODE database (71). A prominent PTBP1 eCLIP peak near the 3′ splice site in the upstream intron of *Ptbp2* exon 10 was detected (Figure 5H). By performing RIP-qPCR assay in SW480 colon cancer cells, we found that PTBP1 indeed binds near this 3′ splice site (Figure 5I), suggesting that PTBP1 directly represses *Ptbp2* exon 10 inclusion in these cells.

Given that PTBP2 is known to induce a neuronal-like splicing program in the brain, we next analyzed whether upregulated PTBP2 induced similar splicing events in crypt cells. We found that among all skipped exons that showed significant splicing changes in PTBP1-deficient crypt cells, 69 events were reported to be regulated either by PTBP1 or PTBP2 in the brain (Figure 5J, detailed in Supplementary Table S10) (33). Among these 69 exons, 85.51% were annotated to be specifically regulated by PTBP2, 4.35% specifically regulated by PTBP1 and 10.14% regulated by both PTBP1 and 2 (Figure 5J). This finding suggests that the induction of neuronal-like splicing patterns in PTBP1-deficient crypt cells is at least in part due to upregulated PTBP2.

### PTBP1 downregulates PHLDA3 and maintains AKT activity in the crypt cells

**Figure 6.**
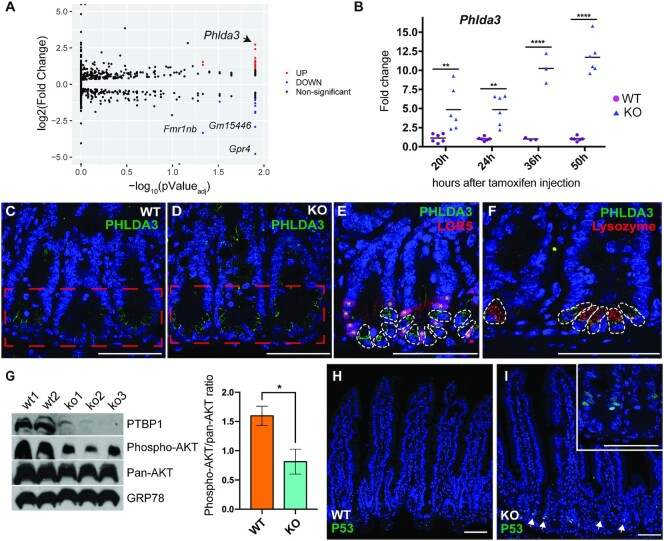
Loss of PTBP1 upregulates PHLDA3 and impairs AKT activation in crypt cells. (**A**) Volcano plot demonstrates mRNA abundance changes in the knockout crypt cells. Red dots represent significantly upregulated genes (log_2_FC > 1, FDR < 0.05), blue dots represent significantly downregulated genes (log_2_FC < 1, FDR < 0.05), and the black dots represent genes with no significant changes in expression between the knockout and control crypt cells. *Phlda3* showed the highest fold change among the upregulated genes (arrow). (**B**) Real-time PCR shows significant upregulation of *Phlda3* mRNA level at 20, 24, 36 and 50 h PTI. Data were normalized to *Gapdh*. Each symbol in the graph indicates a fold change of *Phlda3* expression in individual mice. Bars show mean values. At each time point, sibling littermates were used for analysis. Data shown are representative of at least three independent experiments. (**C**, **D**) Immunofluorescence staining using an anti-PHLDA3 antibody shows that PHLDA3 protein expression is increased in the bottom crypt cells in the knockout mice (D). Dotted boxes show the crypt bottom region. (**E**) Double immunofluorescence staining using anti-PHLDA3 and anti-GFP antibodies shows PHLDA3 protein is localized in cells adjacent to LGR5-positive CBC stem cells (*) in wild-type mice. PHLDA3-positive cells are delineated by white dotted lines. (**F**) Double immunofluorescence staining using anti-PHLDA3 and anti-lysozyme antibodies shows that PHLDA3 protein is localized in Paneth cells in wild-type mice. Crypt cells positive for PHLDA3 and lysozyme are delineated by white dotted lines. (**G**) Western blot shows decreased AKT phosphorylation on ser473 in knockout crypt cells at 24 h PTI. Quantification of the ratio of Phospho-AKT to Pan-AKT is shown in the right panel. Mice used in the assay are littermates. PTBP1 was efficiently depleted in all three knockout mice. (**H**, **I**) Immunofluorescence staining with an anti-P53 antibody shows an upregulation of P53 in the knockout crypt cells at 48 h PTI as indicated by nuclear localization of P53 (inset and arrows in I). ** *P* < 0.01; **** *P* < 0.0001. WT, wild-type; KO, knockout. Scale bars, 50 μm.

Given that loss of PTBP1 caused crypt cell apoptosis, we analyzed genes associated with cell apoptosis among those that are alternatively spliced or differentially expressed upon loss of PTBP1. This led to the identification of *Phlda3*, a gene that encodes a negative regulator of AKT signaling activity. *Phlda3* showed the highest fold change among the upregulated genes in PTBP1-deficient crypt cells (Figure 6A, Supplementary Table S6). We performed real-time PCR to assess the expression of *Phlda3* in PTBP1-deficient crypt cells at different time points after tamoxifen induction. An increase in *Phlda3* expression was first detected at 20 h PTI in PTBP1-deficient crypt cells and became more prominent at 36 and 50 h PTI (Figure 6B). Immunostaining revealed that PHLDA3 protein was highly accumulated in the cells at the crypt bottom where ISCs and Paneth cells are located (Figure 6C and D). In the PTBP1 knockout mice, PHLDA3 protein expression is significantly increased in the crypt bottom region (Figure 6D). To determine which cell lineages express PHLDA3 protein, we performed double immunofluorescence staining with anti-PHLDA3 and anti-GFP antibodies on intestines of non-tamoxifen treated *Ptbp1*^f/f^;*Lgr5*^ER+/−^ mice in which LGR5-expressing CBC stem cells are labeled with GFP. Interestingly, we found PHLDA3 protein is localized in cells that are interspersed between LGR5-positive CBC stem cells (Figure 6E). Double immunofluorescence staining with anti-PHLDA3 and anti-lysozyme antibodies indicates that these PHLDA3-expressing cells are Paneth cells (Figure 6F).

Since PHLDA3 can interfere with AKT binding to PIP_3_ and thereby prevent AKT activation (24), we assessed AKT phosphorylation in PTBP1-deficient crypt cells. Indeed, phosphorylation of AKT on Ser473 was significantly decreased in PTBP1-deficient crypt cells at 24 h PTI (Figure 6G). This was accompanied by an increase in P53 activity at 48 h PTI, judged by the nuclear localization of P53 in the crypt cells (compare Figure 6H and I). Since *Phlda3* is a target of P53 (24,25), we examined whether P53 activation occurred before the upregulation of *Phlda3*. Interestingly, although the mRNA level of *Phlda3* was upregulated significantly in PTBP1-deficient crypt cells at 24 h PTI, we did not detect any P53 activity at this time point (Supplementary Figure S7). This result suggests that upregulation of the *Phlda3* mRNA level in PTBP1-deficient crypt cells at 24 h PTI is induced by a mechanism independent of P53.

**Figure 7.**
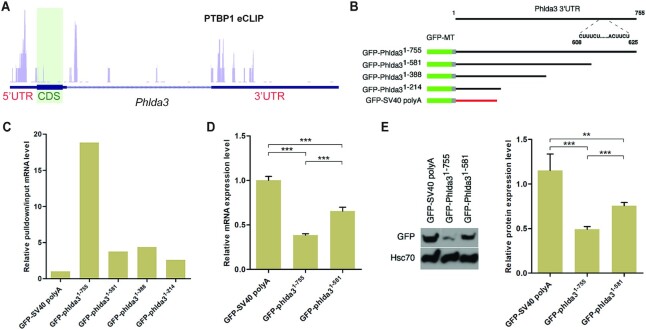
PTBP1 binds to the CU-rich region in the 3′ UTR of *Phlda3* mRNA. (**A**) UCSC track visualization of PTBP1 eCLIP data from HepG2 cells demonstrates PTBP1 binding sites at the UTR regions of the *Phlda3* transcript. CDS, coding sequences. (**B**) Full-length and the truncated *Phlda3* 3′UTR regions were fused to a GFP reporter to generate reporter constructs for the *in vitro* binding assay. A CU-rich region between the positions of 608 and 625 of *Phlda3* 3′UTR is only included in the full-length 3′ UTR reporter construct. (**C**) *In vitro* RIP-qPCR assay demonstrating PTBP1 has a high binding affinity to *Phlda3* 3′UTR when the CU-rich region is present. The data shown are representative of three independent experiments. (**D**, **E**) Cell transfection assay demonstrating this CU-rich region represses the expression of the GFP reporter at both the mRNA (D) and protein level (E) in SW480 cells.

To determine if *Phlda3* mRNA levels in the crypt cells are directly regulated by PTBP1, we analyzed the HepG2 PTBP1 eCLIP data from ENCODE database (71). While no PTBP1 binding was detected in the coding region of *Phlda3*, we identified eCLIP tags at both the 5′ and 3′ untranslated regions of the *Phlda3* transcript (Figure 7A). We next performed the *in vitro* RIP-qPCR assay to determine if PTBP1 directly binds to the 3′UTR region of *Phlda3*. We detected a high binding affinity of PTBP1 to the full-length *Phlda3* 3′ UTR that includes a 17 bp long CU-rich region in the 3′ end (Figure 7B and C). Deletion of this CU-rich region abolished PTBP1 binding (Figures 7B and C). Furthermore, the full-length *Phlda3* 3′ UTR containing this CU-rich region decreases the mRNA and protein levels of a GFP reporter in SW480 cells (Figure 7D to E). Deletion of this CU-rich region increased the expression level of GFP transcripts and protein (Figure 7D to E). These results suggest that PTBP1 directly binds to a CU-rich region in *Phlda3* 3′ UTR, and this region is essential for downregulating the *Phlda3* mRNA and protein expression.

**Figure 8.**
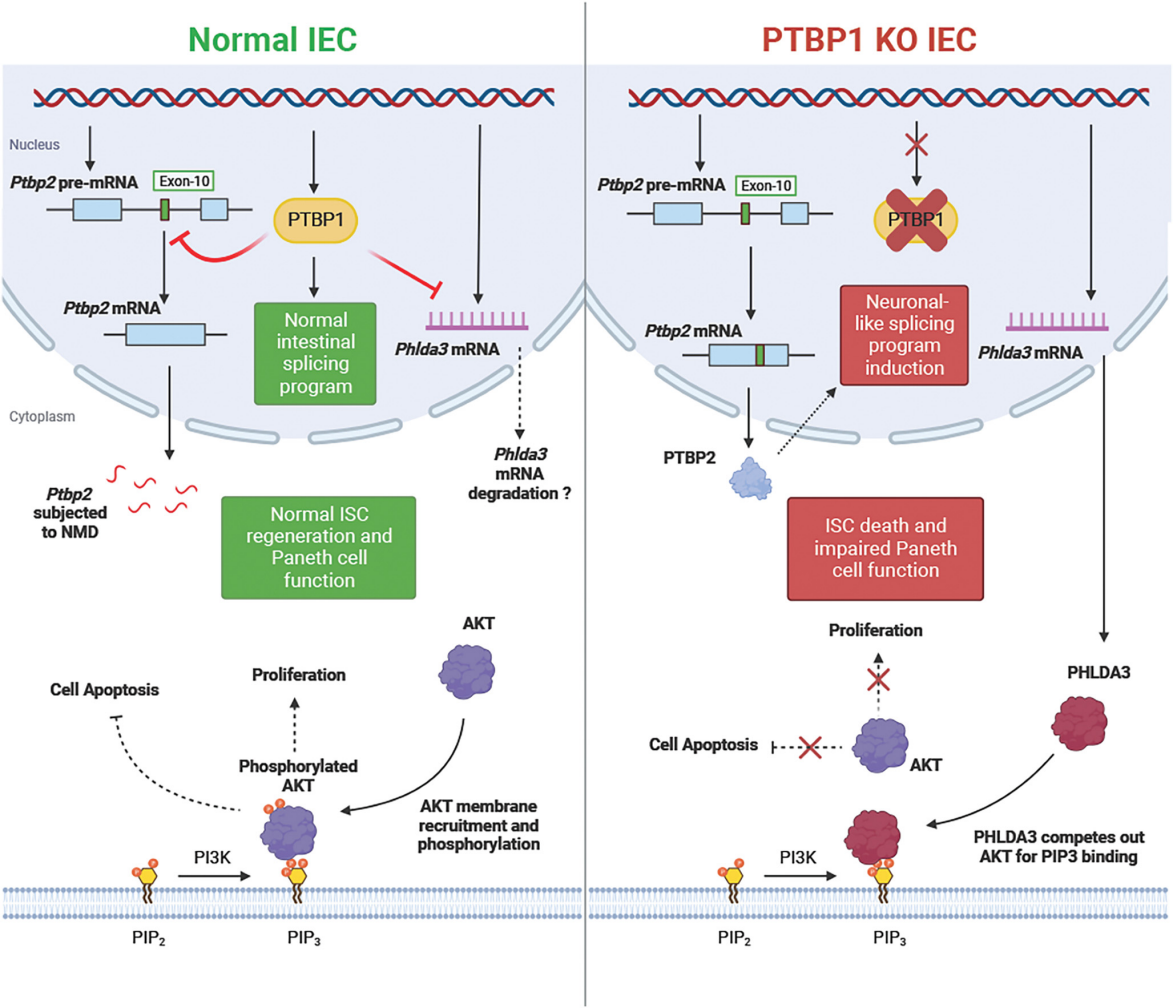
A proposed model for PTBP1 in regulating intestinal epithelial regeneration. PTBP1 maintains ISC survival, proliferation, and stemness through post-transcriptionally regulating *Phlda3* and *Ptbp2* expression. PTBP1 inhibits *Phlda3* presumably by destabilizing its mRNA, which permits AKT phosphorylation and subsequent activation to promote ISC survival and proliferation and sustain Paneth cell plasticity. PTBP1 represses the inclusion of *Ptbp2* exon 10, leading to nonsense-mediated mRNA decay (NMD) of *Ptbp2*, which in turn prevents induction of the neuronal-like splicing program to maintain ISC stemness. Figure was made in biorender.

Collectively, our results reveal a novel mechanism through which PTBP1 post-transcriptionally regulates gene function to support ISC survival and epithelial regeneration (Figure 8). PTBP1 represses PTBP2 expression by promoting its non-productive splicing and suppresses the aberrant execution of a neuronal-like splicing program in the crypt cells, which is probably essential for maintaining ISC stemness. Moreover, PTBP1 downregulates PHLDA3 expression in the Paneth cells and permits AKT activation, which presumably sustains the stem cell regeneration capacity and Paneth cell plasticity.

## DISCUSSION

The intestinal epithelial regeneration is vital not only for digestion and absorption but also for protection against insults from environmental hazards. Under homeostatic conditions, the regeneration of intestinal epithelium is driven by ISCs. Paneth cells are reported to be important in providing a niche that supports ISC survival and self-renewal (4–8). Paneth cells are also capable of acquiring stem cell properties and contributing to epithelial regeneration upon loss of ISCs (10–12). A better understanding of mechanisms by which ISC niche and Paneth cell plasticity are properly maintained will provide novel insight into the intestinal epithelial regeneration process.

AKT is crucial for ISC survival and proliferation (13–16). AKT is also important for Paneth cells to acquire stemness in response to tissue damage (11). Yet, the mechanisms that control AKT activity in the intestine remain largely unclear. We report here that PTBP1, an RNA-binding protein that controls gene function post-transcriptionally, is critical for regulating AKT signaling. We demonstrate that PTBP1 maintains AKT activity by repressing the Paneth cell-specific expression of PHLDA3. Mechanistically, we show that PTBP1 directly binds to a CU-rich region in the 3′ UTR of the *Phlda3* transcript, which we show is required for downregulating the mRNA and protein levels of a reporter gene. This finding raises the possibility that PTBP1 may inhibit *Phlda3* expression by binding to its 3′ UTR and destabilizing its mRNA. Since PTBP2 also binds to CU-rich regions (29,47,64,65) and is upregulated in the knockout mice, it is possible that upregulated PTBP2 may stabilize *Phlda3* by binding to its 3′UTR. Further studies in PTBP1 and PTBP2 double knockout mice are needed to test these possibilities. The physiological significance of PTBP1-mediated *Phlda3* inhibition in Paneth cells is of great interest. Given that Paneth cells nurture and protect ISCs (4,5,7,11), it is tempting to speculate that such inhibition is essential for Paneth cells to support ISC survival and regeneration. In support of this idea, we found that PTBP1 depletion in the LGR5-positive cells alone was insufficient to provoke ISC death, suggesting an impaired ISC niche may result in their death. The disruption of intestinal epithelial regeneration in the PTBP1 knockout mice also suggests that Paneth cells failed to acquire multipotency and regenerate epithelial cells upon the loss of ISCs. This is probably attributed to impaired AKT activation in Paneth cells as well. A follow-up study using a Paneth cell-specific PTBP1 knockout mouse model will elucidate the role of PTBP1 in Paneth cells.

A number of studies indicate that PTBP1 is required to maintain the multipotency of stem cells and progenitor cells by inhibiting splicing programs that promote cell differentiation (41–44). In the brain, this is through repressing the productive splicing of PTBP2 to prevent activation of neuronal-specific splicing programs (32,34,45–47,72). We show that PTBP1 inhibits *Ptbp2* expression in the intestinal crypt cells through a similar splicing control, which inhibits a neuronal-like splicing program in these cells. This inhibition is likely critical for maintaining the multipotency of intestinal crypt cells. Despite a shift toward neuronal-like splicing patterns in PTBP1-deficient crypt cells, we did not detect the activation of key transcriptional factors that promote neuronal differentiation. This suggests that eliminating PTBP1 alone is insufficient to convert cells into neurons; proper cell context and niche are required for neuronal cell differentiation.

Results from our global transcriptome analysis show that, in addition to *Phlda3* and *Ptbp2*, PTBP1 controls the alternative splicing of many other genes in the crypt cells. Loss of PTBP1 causes both an increase and a decrease in exon inclusion in the crypt cells. The increased exon inclusion events, however, are significantly more than the decreased events, suggesting that PTBP1 functions primarily as a repressor of alternative spliced exons in the crypt cells. This is consistent with previous findings that PTBP1 either represses or promotes exon inclusion of alternative exons depending on its binding sites in the pre-mRNAs (29). Using exon ontology, we further show that PTBP1-regulated alternative exons are important for protein structure, localization, modification, binding with other proteins, and catalytic activity. Interestingly, we found these exons are significantly enriched for regions encoding phosphorylation sites, suggesting that PTBP1 regulates the functions of its target genes through the control of their protein phosphorylation. Our gene ontology analysis further revealed that PTBP1-mediated splicing control is important for modifying genes that control RNA splicing, and some of them are reported to be involved in regulating stem cell multipotency. For example, MBNL2, a muscleblind-like RNA binding protein that negatively regulates the splicing program important for pluripotency of embryonic stem cells (73), is aberrantly spliced in PTBP1-deficient crypt cells. While our findings support the idea that aberrant upregulation of *Phlda3* and *Ptbp2* plays an important role in causing ISC death and epithelial regeneration failure in PTBP1-deficient mice, we cannot exclude the possibility that mis-splicing of other genes provoked by PTBP1 deficiency may also contribute to these defects. Follow-up studies are required to determine the contribution of these genes in regulating intestinal epithelial regeneration.

Our studies using the constitutive *Villin* promoter linked-*Cre* mouse model and the tamoxifen-inducible *Cre* mouse model for PTBP1 deletion indicate that PTBP1 plays age-related roles in IECs (39). In the neonatal stage, we showed that PTBP1 deletion mediated by the constitutive *Villin* promoter linked-*Cre* recombinase does not result in epithelial regeneration defects in the small intestine (39). Instead, the knockout mice develop intestinal inflammation in the colon shortly after birth, which is accompanied by early onset of colitis and colorectal cancer (39). Mechanistically, we demonstrate that epithelial PTBP1 downregulates the Toll-like receptor signaling activity to suppress the intestinal immunity in the neonates, which is important for generating a permissive environment for gut microbiota formation (39). The discrepancy in PTBP1 functions at the neonatal stage and adulthood are likely due to the differences in epithelial regeneration mechanisms between the two stages. Unlike the adult small intestine wherein Paneth cells support ISC survival and proliferation, the neonatal intestine is immature and Paneth cells do not appear in the neonatal intestine until the second week after birth (74,75). As such, a Paneth cell-independent mechanism accounts for the ISC survival and regeneration in the early neonatal stage (76). In addition, the neonatal period is a critical time window for microbial colonization which requires transient intestinal immune suppression (77). The physiological role of PTBP1 in the neonatal intestine is to presumably mediate immune suppression instead of regulating ISC survival and proliferation.

## DATA AVAILABILITY

All raw RNA-seq data files are available for download from the Gene Expression Omnibus (accession number GSE185499).

## Supplementary Material

gkad042_Supplemental_Files
