# Supplementary material for: PTBP1 controls intestinal epithelial regeneration through post-transcriptional regulation of gene expression
Source: Nucleic Acids Res. 2023 Feb 6;51(5):2397–414. doi: 10.1093/nar/gkad042 (PMC10018364; doi:10.1093/nar/gkad042)
Supplement: gkad042_Supplemental_Files [file gkad042_supplemental_files.zip › Supp figures.pdf]

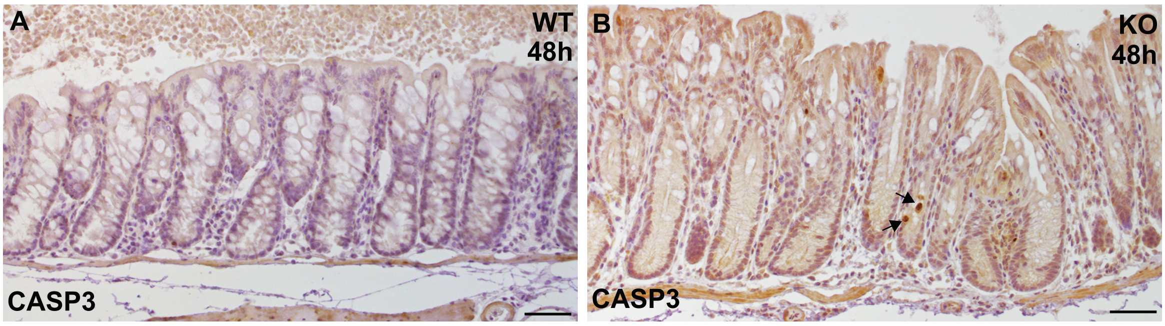

Figure S1. Loss of PTBP1 in intestinal epithelial cells causes apoptosis in the colon
(A-B)) Immunohistochemical staining with an anti-active CASPASE3 antibody shows an increase in the number of apoptotic cells in the colon of *Ptbp1*$^{f/f}$;*Vil-cre*$^{ER+/-}$ (KO) mice at 48 PTI (arrows in B) when compared to the *Ptbp1*$^{f/f}$ (WT) mice. Scale bars, 50 µm.

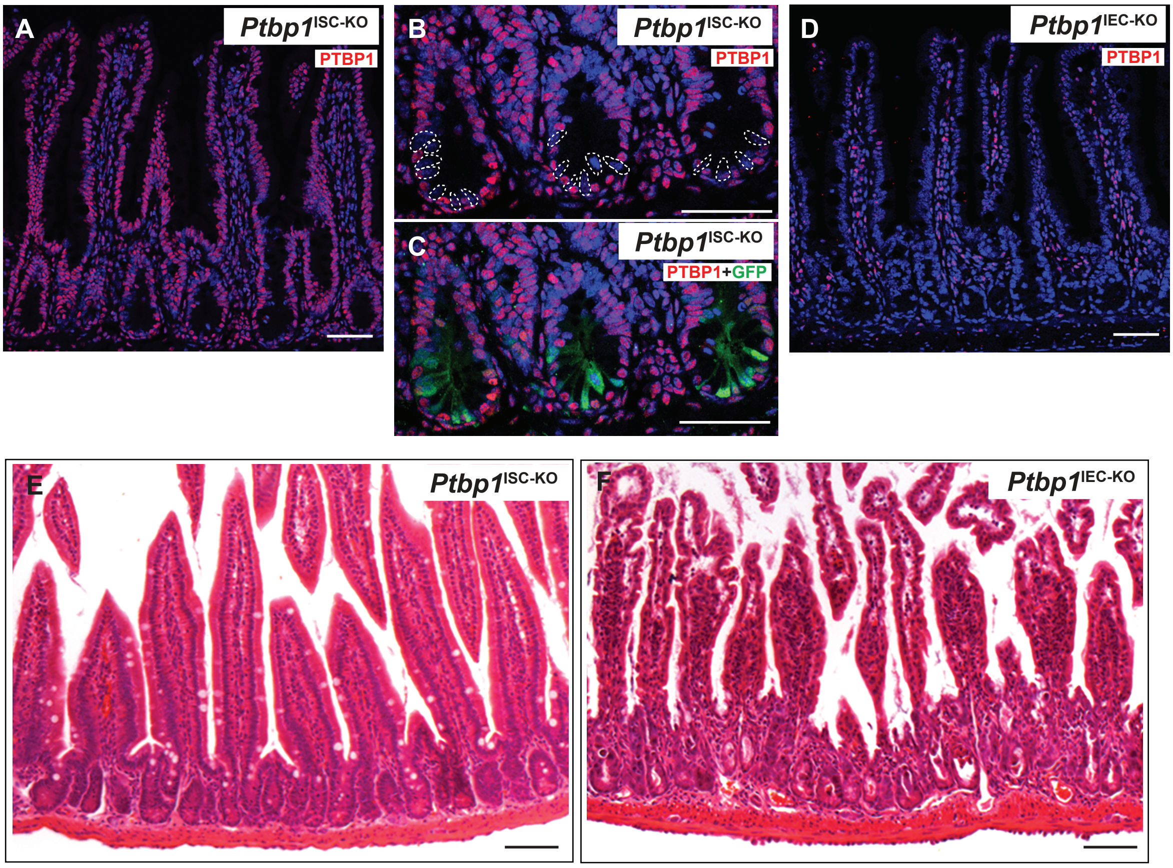

Figure S2. Loss of PTBP1 in *Lgr5*-expressing ISCs does not cause ISC apoptosis
(A-D) Immunofluorescence staining with anti-PTBP1 and anti-GFP antibodies shows differential PTBP1 deletion in tamoxifen-administrated *Ptbp1*$^{f/f}$;*Lgr5* $^{ER+/-}$ mice (denoted by *Ptbp1*$^{ISC\text{-}KO}$) and *Ptbp1*$^{f/f}$;*Vil-cre*$^{ER+/-}$;*Lgr5*$^{ER+/-}$ mice (denoted by *Ptbp1*$^{IEC\text{-}KO}$) at 48 PTI. Note that PTBP1 expression is diminished in the entire IECs but remains intact in the lamina propria cells of *Ptbp1*$^{f/f}$;*Vil-cre*$^{ER+/-}$;*Lgr5*$^{ER+/-}$ mice (D), whereas in in *Ptbp1*$^{f/f}$;*Lgr5* $^{ER+/-}$ mice, PTBP1 is only deleted in GFP-positive *Lgr5*-expressing ISCs (A, B, and C). Crypt cells that lack PTBP1 protein expression are delineated by white dotted lines (B). (E-F) Hematoxylin and eosin –stained sections show destruction of the epithelium structure and death of crypt cells in *Ptbp1*$^{f/f}$;*Vil-cre*$^{ER+/-}$;*Lgr5*$^{ER+/-}$ mice (F), which were not detected in *Ptbp1*$^{f/f}$;*Lgr5* $^{ER+/-}$ mice at 72 hours PTI (E). Scale bars, 50 µm.

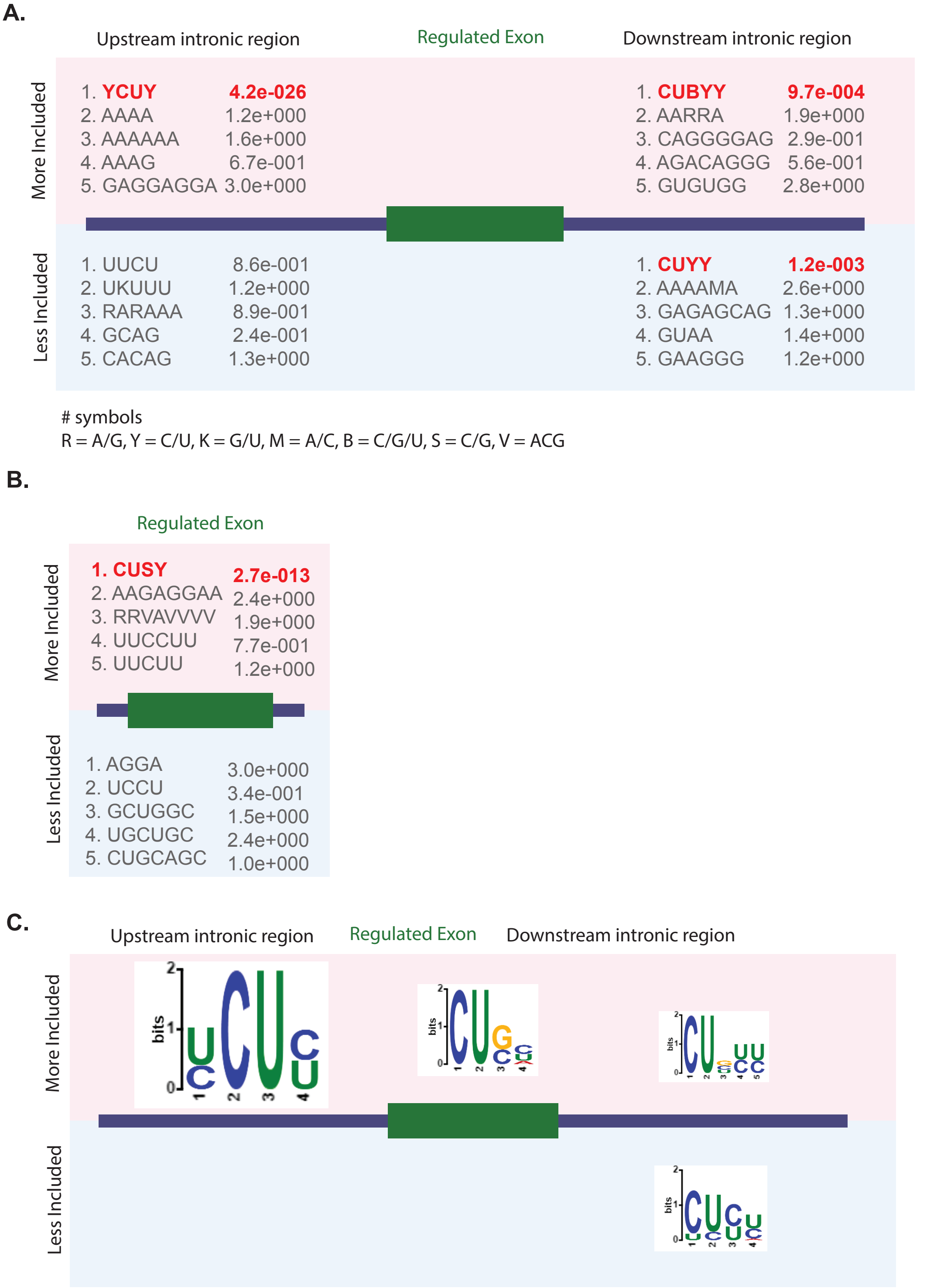

Figure S3. De-novo motif discovery around regulated cassette exons. MEME-based motif discovery in and around cassette exons that are differentially spliced in PTBP1-deficient crypt cells. Top motifs enriched in upstream and downstream regions around regulated cassette exons (A) and within regulated cassette exons (B). E-values are given adjacent to each motif denoting statistical significance. Significant motifs are highlighted in red bold characters. (C) Summary of significantly enriched motifs proximal to regulated exons. YCUY motif in the upstream intronic region of exons with increased inclusion show the most prominent enrichment.

A. Upstream intronic region

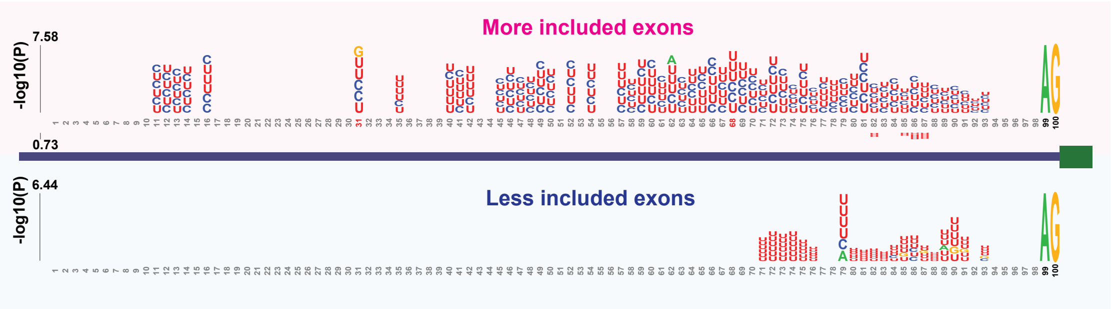

B. Downstream intronic region

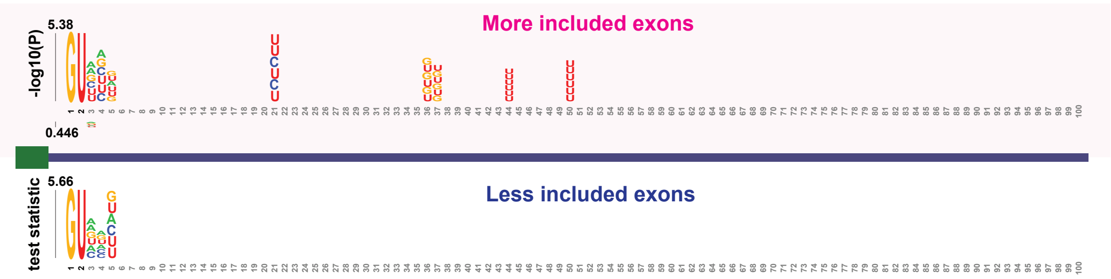

Figure S4. Position specific k-mer enrichment proximal to regulated cassette exons. Unbiased k-mer probability logo analysis showing position-specific enrichment of all hexameric motifs proximal to regulated cassette exons in the upstream (A) and downstream intronic region (B). Striking enrichment of CU-rich sequences is observed in the upstream intronic region of exons that increase their inclusion upon PTBP1 depletion.

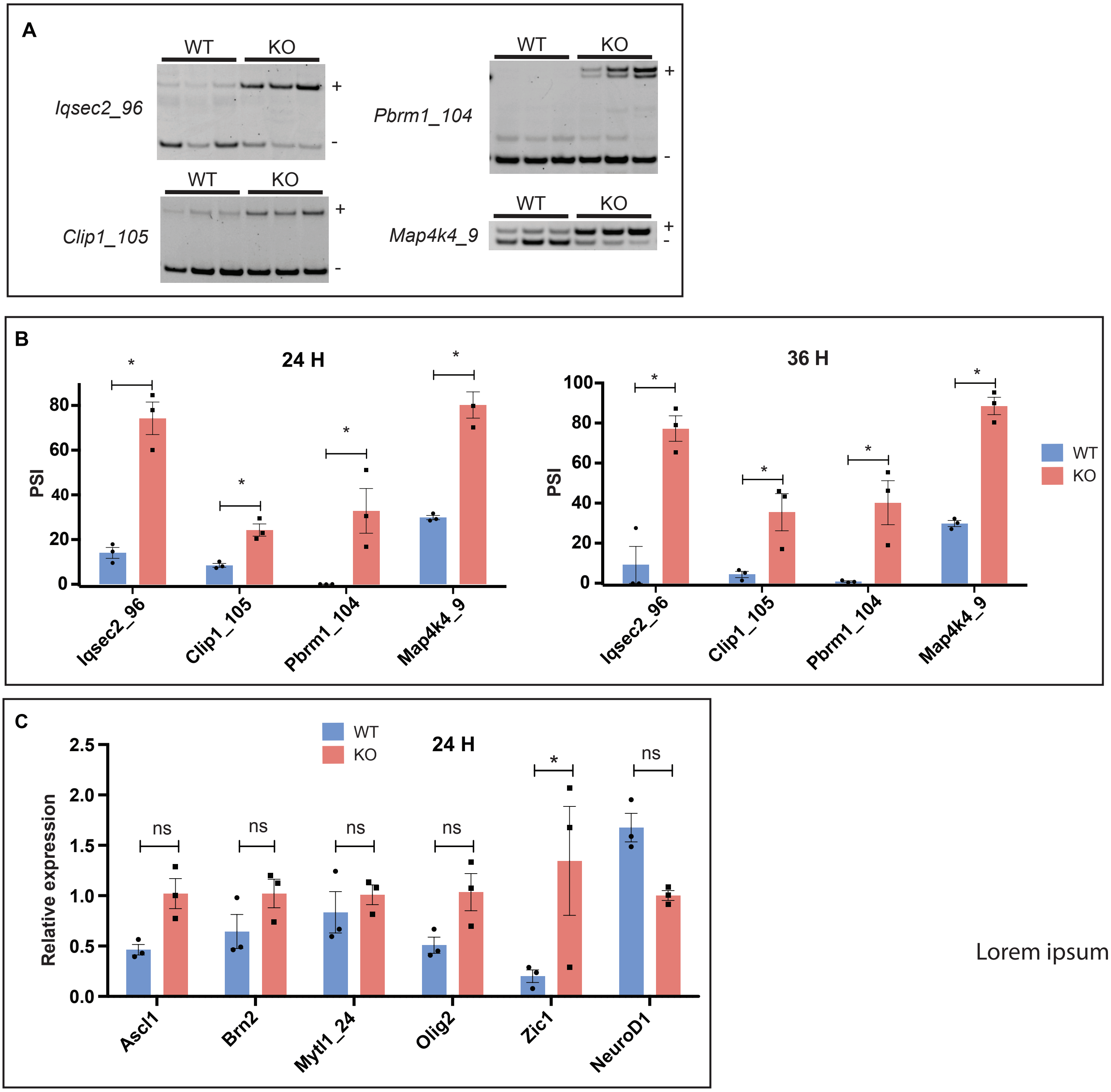

Figure S5. Loss of PTBP1 alters neuronal gene splicing in the crypt cells.
(A-B) PCR-based splicing assay demonstrates splicing changes in genes related to neuronal differentiation. Gel images show a significant increase in exon inclusions upon PTBP1 depletion (A). Exon inclusion and exclusion bands are denoted by (+) and (–) respectively. Quantification was done using Image Lab 5.2.1 software (Biorad). PSI values were determined by using ImageLab software (BioRad) as the exon inclusion band intensity/(the exon inclusion band intensity + the exon exclusion band intensity) x 100. Multiple t tests were performed using Holm-Sidak method, with alpha=5% and without assuming consistent SD (B). (C) qRT-PCR analysis of critical transcription factors known to promote neurogenesis. Multiple t tests were performed using Holm-Sidak method, with alpha=5% and without assuming consistent SD.

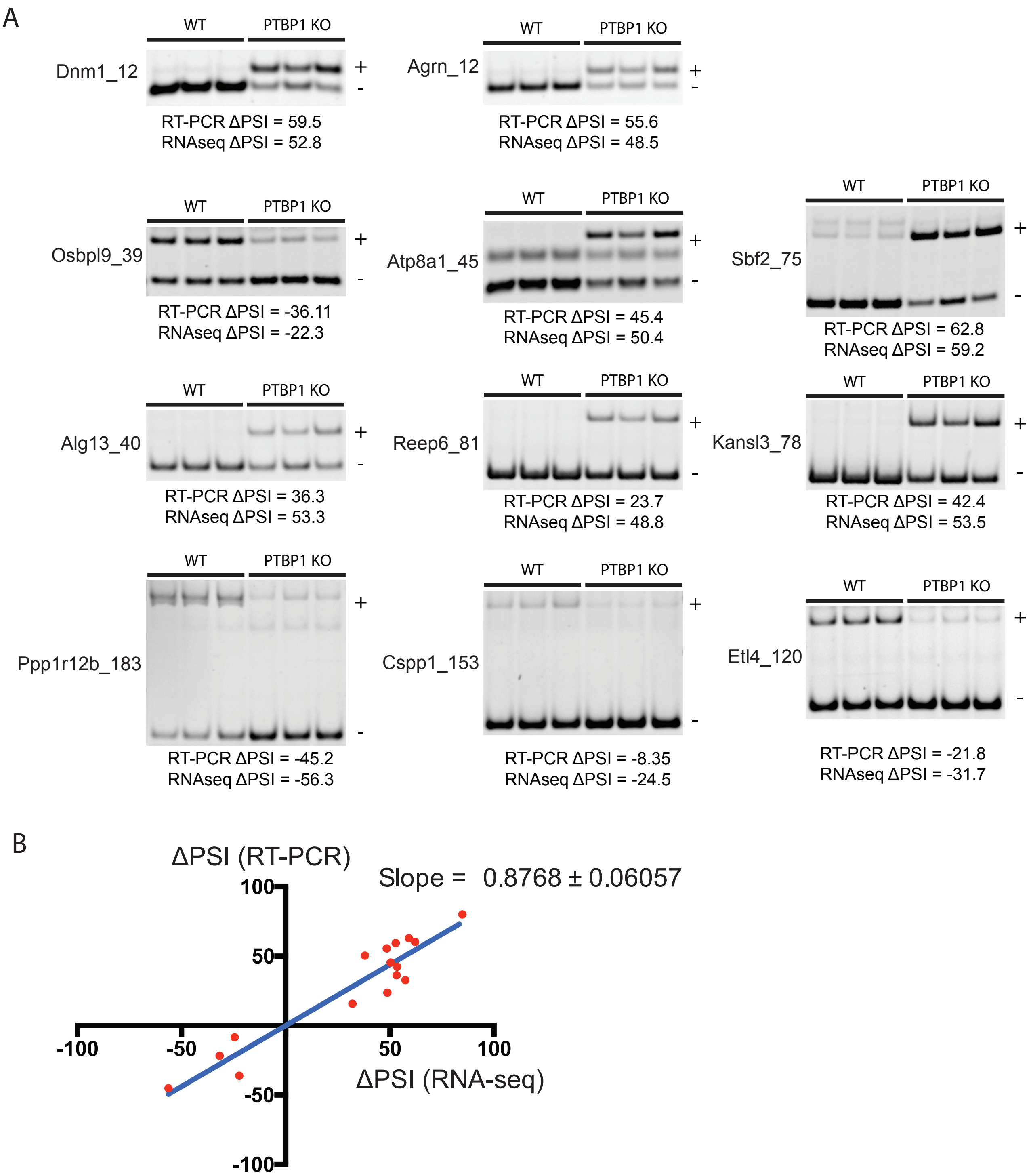

Figure S6. Validation of 11 alteraltive splicing events in PTBP1-deficient crypt cells
(A) PCR-based splicing assays show validation of 11 splicing events identified by RNAseq analysisin. Exon inclusion and exclusion bands are denoted by (+) and (–) respectively. (B) Scatterplot comparing Δ PSI values determined using RNA-seq analysis and gel-based RT-PCRs. RT-PCR Gel quantification was done using Image Lab 5.2.1 software (Biorad). PSI values were determined as [100 X exon inclusion band intensity/ (the exon inclusion band intensity + the exon exclusion band intensity)] and ΔPSI values were computed as the difference between mean PSI values of KO and WT IECs.

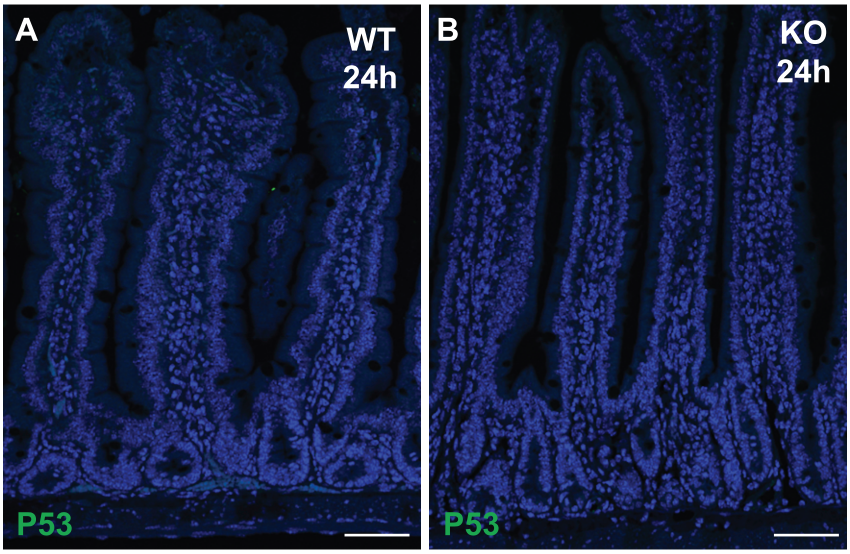

Figure S7. P53 is not upregulated in the PTBP1-deficient crypt cells at 24 hours PTI. (A-B) Immunofluorescence staining with an anti-P53 antibody shows no detectable P53 activity in wild-type or PTBP1-deficient crypt cells at 24 hours PTI. WT, wild-type; KO, knockout. Scale bars, 50 µm.
