# Supplementary material for: PTBP1 controls intestinal epithelial regeneration through post-transcriptional regulation of gene expression
Source: Nucleic Acids Res. 2023 Feb 6;51(5):2397–414. doi: 10.1093/nar/gkad042 (PMC10018364; doi:10.1093/nar/gkad042)
Supplement: gkad042_Supplemental_Files [file gkad042_supplemental_files.zip › Supp tables.pdf]

Supplementary Table 1: Primer sequences for genotyping

gene	Forward (5' to 3')	Reverse (5' to 3')
Ptbp1 floxed allele	CCCATAACTGTCCATAGACC	TGTTGGTAATGCCAGCACAG
Villin-creERT2	GTGTGGGACAGAGAACAAACCG	TGCGAACCTCATCACTCGTTGC
Lgr5-creERT2	CTGCTCTCTGCTCCCAGTCT	CTGAACTTGTGGCCGTTTAC

Supplementary Table 2: Primer sequences for qPCR

gene	Forward (5' to 3')	Reverse (5' to 3')
Ptbp1	TCTACCCAGTGACCCTGGAC	GAGCTTGGAGAAGTCGATGC
Phlda3	TTCACGCTAGTGACCGAAGG	ACAGTCTGGATGGCCTGTTG
Ptbp2	TCCCTACTAGCTGTTCCAGGG	GGGGCGTAACCATCTCTTCA (in exon 10)
Ptbp2	ACCAGGCATTTTTGGAACTG	TGTGGTGCCACTAAGAGGTG
Lgr5	CCTACTCGAAGACTTACCCAGT	GCATTGGGGTGAATGATAGCA
Ascl2	CTACTCGTCGGAGGAAAG	ACTAGACAGCATGGGTAAG
Smoc2	GACCCCTCTTCCTCTTCTGG	TCCTTCTTGCCAATGTCTCC
Lrig1	TAGAGGAGTGGCATCTGGAG	CCATGCGCTAAGGATTAAAA
Hopx	CTTCAACAAGGTCAACAAGCAC	AGGCGCTGCTTAAACCATT
Gapdh	TTCTTGTGCAGTGCCAGCC	CACCGACCTTCACCATTTTGT
Ascl1	CATCTCCCCCAACTACTCCA	CCAGCAGCTCTTGTTCCTCT
Brn2	GCGGATCAAACTCGGATTTA	TCTGCCTCTTCCAACCACTT
Mytl1	ATCAAGCCATGGAAACTTGG	TCCACCTCTGACAAGCTCCT
NeuroD1	CAAAGCCACGGATCAATCTT	TCCCGGGAATAGTGAAACTG
Olig2	ATGCACGACCTCAACATCGCCA	ACCAGTCGCTTCATCTCCTCCA
Zic1	TTTCCTGGCTGCGGCAAGGTTT	ACGTGCATGTGCTTCTTGCGGT
GFP	CTGAAGTTCATCTGCACCAC	GTCCTTGAAGAAGATGGTGC

Supplementary Table 3: Primer sequences for splicing assay

gene	Forward (5' to 3')	Reverse (5' to 3')
Ptbp2 exon 10	TGGCTATTCCAAATGCTGCT	TCCCATCAGCCATCTGTATC
*Clip*1_105 bp	GGACCTTCAGTTCCGAGTTG	CTCTCGAGCTCCGAAGTGTT
*Iqsec*2_96 bp	TGGGACAGGTCCAGACTCAT	GATGCTTGCCCATCTGGTT
*Map4k4*_9 bp	AGCCAAAAAGCCTGACGATA	CTGGTCCCGAGGTAGAATCA
*Pbrm1*_104 bp	GCCAACACTTGGTGTCACAT	GACACTGCTGGAAGGAGAGG
*Sbf2*_75 bp	TCAGAAACTCAGAGGCAGCA	CTGAAGGAGGCTGAGTGGTC
*Kansl3*_78 bp	CTCCCAGCTGCTGAAAAGAC	AAGAGTGCAAGGTCCTCCAA
*Alg13*_40 bp	GTGGCAGCACCACTACCACT	CTGCAATCCCAGATTGAAATAG
*Dnm1*_12 bp	TCAGCAGAGAAGCAACCAGA	ACTCCTTGGAGCCTCCCTTC
*Atp8a1*_45 bp	CAAAACTGGGACCCTGACAT	CAACGACGGGTCATTAAAGG
*Reep6*_81 bp	GCACCACATGGCTCTAGACAG	TGGTTGCAGTGGTGTCTTTG
*Agrn*_12 bp	GAGCAAAGAGCCCATAGCC	ATAGAGGGGCTCCTTGAGGT
*Cspp1*_153 bp	TTCGGGAAAGAGAAGCAAGA	GGCTCACCAAATGTGTTTCC
*Etl4*_120 bp	GCCAACACCACAGACTAGAGC	GGGCCATTTTCACATTCAAC
*Osbpl9*_39 bp	TTAAACACTGCATTGTGTTGCT	CTGGAGGTAAGGCAGTCTGG
Ppp1r12b 183 bp	GGATGAAGCAAAGGGAAGTG	CTCCACAGAGGGGTCTGTTC

Supplementary Table 4: Primer sequences for RIP assay

gene	Forward (5’ to 3’)	Reverse (5’ to 3’)
Ptbp2	ACCAACTTGCCCCAATTAAC	GGAAGAGCGGAGATGAACAG

Supplementary Table 5. List of significantly alternated splicing events.
Differential splicing analysis was performed using rMATS v4.0.2(turbo), and events with FDR < 0.1, junction read counts ≥ 10, |ΔPSI| ≥ 10% were deemed to be significant. A3SS: alternative 3’ splice site, A5SS: Alternative 5’ splice site, MXE: Mutually exclusive exon, RI: Retained intron, SE: Skipped exon.

Supplementary Table 6. List of genes that showed significant differential expression.
Differential gene expression events were assessed using the Cufflinks package, and events with FDR(q-value) < 0.05, FPKM≥1, and log2(fold change) ≥ 1 were identified as significant.

Supplementary Table 7: List of alternative splicing events categorized as involved in RNA splicing regulation according to gene ontology.

Supplementary Table 8: List of neuronal-related genes that showed significant splicing changes in PTBP1-deficient crypt cells.

Supplementary Table 9: List of genes that showed over 1.5-fold change in gene expression.
Differential gene expression events were assessed using the Cufflinks package, and events with FPKM≥1, q-value ≤ 0.2, and |log2 (fold change)| ≥0.585 were identified as significant.

Supplementary Table 10: Alternative splicing events in PTBP1-deficient crypt cells specifically regulated by PTBP1/PTBP2. This table describes 69 skipped exon events that showed significant splicing changes in PTBP1-deficient crypt cells and were reported to be regulated specifically either by PTBP1 or PTBP2 in the brain.
